# High sensitization to *Rhizopus nigricans* in children with allergic asthma in Southwest China: A microfluidic chip and proteomics study^☆^

**DOI:** 10.1016/j.waojou.2025.101097

**Published:** 2025-08-08

**Authors:** Heping Fang, Juan Li, Xiangyu Li, Xiang Wen, Dan Zeng, Yuyi Tang, Run Wang, Na Zang, Wen Zhong, Luo Ren, Enmei Liu

**Affiliations:** a Department of Respiratory Medicine, Children's Hospital of Chongqing Medical University, National Clinical Research Center for Child Health and Disorders, Ministry of Education Key Laboratory of Child Development and Disorders, Chongqing Key Laboratory of Child Rare Diseases in Infection and Immunity, Key Laboratory of Children's Important Organ Development and Diseases of Chongqing Municipal Health Commission, Chongqing, China; b Guangzhou National Laboratory, Guangzhou, China; c Department of Allergy, Chongqing General Hospital, Chongqing, China

**Keywords:** Allergic asthma (AA), Immunoglobulin E, Fungi, *Rhizopus nigricans* (RN), Children

## Abstract

**Background:**

Fungi play a significant role in promoting acute exacerbations and poor control of allergic asthma (AA), particularly in children. In Southwest China, characterized by a humid and warm climate, moderate year-round fungal pollution is closely associated with the onset of childhood asthma. Despite this, little is known about the characteristics of fungal allergens in asthmatic children in this region.

**Objective:**

This study aimed to investigate fungal sensitization in children with AA and its association with AA features.

**Methods:**

A cross-sectional study was conducted at a children's hospital in Southwest China, involving 281 AA children and 20 healthy controls. Specific IgE (sIgE) levels for 13 fungal species and 3 *Staphylococcal enterotoxins* (SEs) were measured using microfluidic chips. Olink proteomics was used to analyze plasma samples from 46 AA children to explore molecular features associated with fungal sensitization.

**Results:**

The sensitization rate to fungi and SEs in AA children was 75.4% (compared to 30% in healthy controls), with *Rhizopus nigricans* (RN) showing the highest sensitization rate at 67.3% (0% in healthy children). RN-sIgE was significantly correlated with total IgE (*Rho* = 0.76), *D. pteronyssinus*-sIgE (*Rho* = 0.67), *D. farinae*-sIgE (*Rho* = 0.67), blood eosinophil count (BEC, *Rho* = 0.26), fractional exhaled nitric oxide (FeNO, *Rho* = 0.20), and Childhood Asthma Control Test (C-ACT, *Rho* = −0.17). Proteomic analysis identified 61 upregulated plasma proteins in AA children with RN sensitization, including IL5RA, PRG2, PRSS2 and PRG3, forming a protein-protein interaction (PPI) network linked to innate immunity (53.1%) and proteolysis (21.9%). These proteins showed greater overlap with mycosis-associated proteins in the UK Biobank than with AA-associated proteins. A total IgE threshold of 395.0 kU/L statistically predicted RN sensitization with high accuracy (AUC 0.91) in this population. This was demonstrated in a case of refractory “nonallergic” asthma (negative for 19 common allergens) with an elevated total IgE level (461.4 IU/mL) and RN sensitization (0.72 IU/mL).

**Conclusion:**

RN sensitization is relatively common in children with AA from Southwest China and may be associated with innate immune responses and proteolysis pathways. These findings suggest a possible underappreciation of RN's role in AA, warranting further investigation.

## Introduction

Allergy and asthma are major global health issues recognized by the World Health Organization (WHO). As of 2019, the number of asthma patients worldwide had reached 262 million, with approximately 641,000 deaths attributed to the disease.^1^ Children are more susceptible to asthma than adults, with the highest prevalence observed in the 5–9 year age group.^1^ Allergen exposure significantly impacts asthma control, particularly in children, where allergic asthma (AA) predominates.

Fungal exposure has been identified as a risk factor for acute exacerbations and poor control of AA.^2^ Data from the United States indicate that children and adolescents have the highest rates of fungal sensitization.^3^ In Southwest China, moderate year-round fungal pollution has been linked to the onset of pediatric asthma.^4^^,^^5^ In Northern China, fungal mixtures have become the most common allergens in children between 2010 and 2020, with sensitization rates reported to exceed 40%.^6^ In Germany, the population-wide fungal sensitization rate increased from 1998 to 2007 to 2008–2017.^7^ Furthermore, expanding allergen testing has revealed that many adults with severe non-allergic asthma exhibit detectable specific IgE (sIgE), including fungi and *Staphylococcal* enterotoxins (SEs).^8^ This trend may be linked to changes in fungal exposure resulting from urbanization and lifestyle shifts.^9^

Although more than 25 fungi have been reported to be associated with human allergic diseases,^10^ only a limited number of fungal allergens or mixtures are commonly tested in clinical practices in China.^6^^,^^11^ “Hidden” fungal allergens may trigger unexpected acute asthma exacerbations, such as those observed during thunderstorm asthma events.^12^ Recognizing these challenges, the European Academy of Allergy and Clinical Immunology (EAACI) position paper has emphasized the need for improved epidemiological data and multiplex diagnostic methods for fungal sensitization.^13^ This study aims to investigate fungal sensitization in children from Southwest China and its association with AA features using allergen microfluidic chips and plasma Olink proteomics.

## Methods

### Study design

**Fig. 1:**
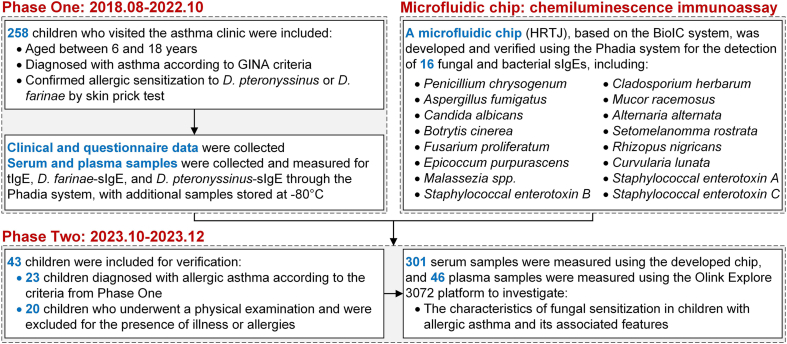
The design and flowchart of this study

This cross-sectional study was conducted at a children's hospital in Southwest China, serving patients mainly from Chongqing, Sichuan, Guizhou and Yunnan Provinces. The study was divided into 2 phases: Phase One (August 2018 to October 2022) included 258 AA children, while Phase Two (October 2023 to December 2023) involved an additional 23 AA children and 20 healthy controls for targeted validation. The one-year interval between phases allowed for internal validation of initial findings within the same environmental context, given the known geographic specificity of allergen sensitization patterns. Blood samples were collected from all participants and analyzed using a customized microfluidic chip to measure sIgE levels against selected allergens, including fungal species and SEs. Subsequent Olink proteomic analysis was performed on 46 AA children with different fungal sensitization profiles to compare plasma protein expression patterns (Fig. 1).

### Ethics approval

This study was approved by the Ethics Committee of our hospital (Nos. 2018-2, 2019-27-1, 2023-102, 2023-103). Written informed consent was obtained from the legal guardians of all children with AA. For healthy children, only residual clinical samples from routine tests were used, and informed consent was waived in accordance with ethical guidelines. All procedures adhered to the Declaration of Helsinki (version 2013).

### Inclusion criteria

The diagnosis of asthma was based on the recommendations of the Global Initiative for Asthma (GINA) 2018–2023. It included typical asthma symptoms that varied over time and in intensity and were associated with triggering factors. Pulmonary function tests demonstrated reversible airway obstruction, and other possible diseases were excluded. Inclusion criteria required participants to be aged 6–18 years, diagnosed with asthma, and confirmed to have dust mite sensitization via a skin prick test. The criterion for dust mite sensitization was established because dust mites are the most common allergen in Southern China.^14^ Thus, AA children sensitized to dust mites represent the majority of AA cases in our study population. It is worth noting that sensitization to dust mites does not exclude the possibility of sensitization to other allergens, such as pollen. The exclusion criteria included other lung diseases, such as bronchopulmonary dysplasia or chronic diseases affecting other organ systems. Healthy controls were required to be aged 6–18 years, with normal physical examination results, no history of allergic diseases, no recorded allergies in the hospital system, and no acute infections in the past 2 weeks.

### Sample collection

Serum and plasma samples were collected and stored at −80 °C in ultralow temperature refrigerators without thawing until analysis. IgE can be stored at −80 °C for long periods with minimal impact from repeated freezing and thawing cycles, maintaining high stability even after 10 years.^15^ The Phadia100 (ImmunoCAP, Uppsala, Sweden) was used to measure total IgE (tIgE), *D. pteronyssinus*-sIgE and *D. farinae*-sIgE at the time of sample collection.

### Data collection

Data were collected and categorized into clinical data and questionnaire data. Clinical data included diagnostic details, the use of inhaled corticosteroids (ICSs), serum tIgE, *D. pteronyssinus*-sIgE, *D. farinae*-sIgE, blood eosinophil count (BEC), the forced expiratory volume in 1 s to the predicted value ratio (FEV1%Pred), and fractional exhaled nitric oxide (FeNO) levels. The questionnaire data included basic demographic information and the Childhood Asthma Control Test (C-ACT), among other data. Notably, implementing the C-ACT for all participants aged 6–18 years aimed to ensure consistency in the evaluation methods and enhance the validity of the statistical analyses (Appendix 1).

### Targeting allergens

Given the extensive diversity of fungi, and constraints in sample testing, technology, and costs, 13 fungal species and 3 SEs (SEA, SEB, and SEC) were selected as detection targets, guided by the literature and the availability of raw materials (Appendix 2). SEs were included due to prior evidence of their association with asthma,^16^ although the primary focus of this study remains fungal sensitization.

### Microfluidic chip

**Fig. 2:**
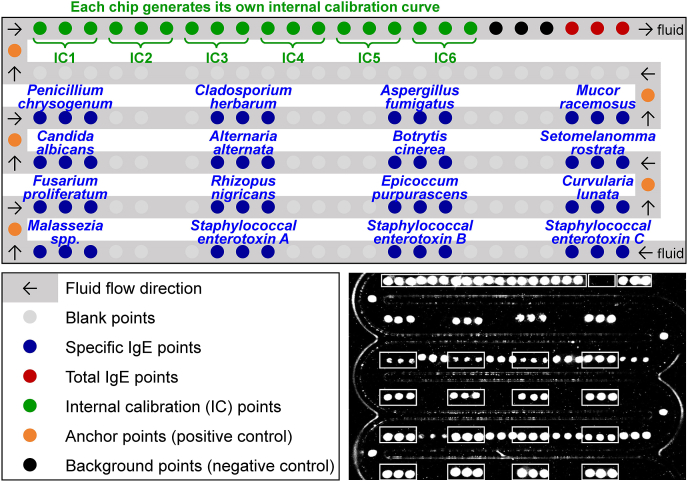
Schematic of allergen extract sample loading positions and fluid flow direction in the microfluidic chip

The microfluidic chip (Fig. 2) used in this study was provided by HRTJ Biotechnology (Xiamen) Co., Ltd., which is developed based on the BioIC system. Previous studies have validated the accuracy of the BioIC system, demonstrating substantial agreement with the Phadia system for most analytes (76.3%–100%).^17^, ^18^, ^19^ The BioIC system is widely used in both clinical practice and research.^20^, ^21^, ^22^ In this study, the accuracy of the chip was further assessed by comparison with the Phadia system, demonstrating 84.6% agreement in sIgE detection (Appendix 3). The Spearman correlation coefficient for tIgE between the 2 systems (HRTJ and Phadia) was 0.75.

### Chip-based IgE measurement

All the serum samples were tested for tIgE, fungal sIgE and SE-sIgE using the microfluidic chip from October to December 2023. The testing instruments and procedures are detailed in Appendix 4. The tIgE standard curve is traceable to the 3rd International Standard for serum IgE established by the World Health Organization. Each chip includes 6 internal calibration points for tIgE, enabling independent internal correction for every test. The sIgE levels were interpolated based on the tIgE calibration curve. In the Phadia system, tIgE is presented in kU/L and sIgE in kU_A_/L. To distinguish, both tIgE and sIgE are presented in IU/mL in the HRTJ system, with a positive threshold of 0.35 IU/mL for sIgE.

### Plasma proteome atlas

Olink proteomic analysis was conducted by Shanghai Sequanta Technologies Co., Ltd. Through extreme-phenotype sampling, we selected 46 AA children comprising 2 subgroups: 23 with the highest combined *Rhizopus nigricans* (RN)-sIgE and total IgE levels (RN-sensitized group) and 23 demographically matched controls with the lowest levels (non-sensitized group). This design enhanced detection sensitivity for proteomic differences given (1) the absence of established clinical thresholds for RN sensitization and (2) the practical constraints of high-throughput proteomics. The general information and clinical characteristics of these children are summarized in Appendix 5. Plasma proteins were assessed using multiplex Proximity Extension Assay technology on the Olink Explore 3072 platform with high-throughput sequencing. Details of the experimental process and data generation are summarized in Appendix 6.

### Sample size and confidence level

The sample size calculation was performed via PASS 15 software. Owing to the scarcity of relevant research, the sample size was based on the initial 20 test results, indicating that a minimum of 264 samples were needed to achieve a 95% confidence level and a 5% allowable error. Upon completion of all tests, a confidence level of 94% was achieved (Appendix 7).

### Statistical and bioinformatic analysis

All statistical analyses and visualizations were performed using R (v4.1.0), GraphPad Prism (v9), and Origin (2021). Missing data with rates below 20% were addressed using multiple imputation (Appendix 8). The Mann-Whitney *U* test was used to compare continuous variables when the data did not meet the assumptions of normality. The chi-square test was employed to assess the association between categorical variables. Fisher's exact test was used when the sample size was small or the expected frequency in any cell of the contingency table was less than 5. Spearman correlation analysis was conducted to evaluate the monotonic relationship between 2 continuous variables. Receiver operating characteristic (ROC) analysis was performed to assess the performance of tIgE levels in identifying fungal sensitization, particularly RN sensitization. The intergroup comparison of the Spearman correlation coefficients was conducted based on the Fisher z-transformation and z test. Sensitivity analyses were conducted to account for potential confounding effects related to group composition.

Bioinformatic analyses included differentially expressed protein (DEP) analysis to identify proteins with significant differences in expression levels between AA children with and without RN sensitization. A protein-protein interaction (PPI) network was constructed to explore the interactions among DEPs. Enrichment analyses based on Gene Ontology (GO) terms and Kyoto Encyclopedia of Genes and Genomes (KEGG) pathways were conducted to understand the biological significance of the DEPs. Specific annotations for DEPs of interest were obtained from the Human Protein Atlas database. Additionally, the plasma proteome atlas from the UK Biobank (covering AA and mycoses) was utilized as a supplementary database in this study.^23^ Statistical significance was defined as *P* < 0.05 for conventional analyses and *P* < 0.01 for bioinformatic analyses.

## Results

### Characteristics of the participants

The characteristics of the 281 AA children are presented in Table 1. The 20 healthy children (9 males and 11 females) had a median age of 8.7 (6.9, 10.1) years, with no significant differences in age (*P* = 0.91) or sex ratio (*P* = 0.23) compared with the 23 AA children in Phase Two.

**Table 1:** The characteristics of the children with allergic asthma.

Characteristics	Overall (*N* = 281)	Phase one (*N* = 258)	Phase two (*N* = 23)	*P* value
Age (year)	8.7 (7.3, 10.8)	8.7 (7.3, 10.7)	8.4 (7.0, 10.9)	0.62
Sex, male	172 (61.2%)	157 (60.9%)	15 (65.2%)	0.82
BMI-for-age Z score	0.39 (−0.58, 1.27)	0.39 (−0.58, 1.27)	0.62 (−0.46, 1.38)	0.48
Region, Chongqing	160 (56.9%)	151 (58.5%)	9 (39.1%)	0.08
Family history of allergies	150 (53.4%)	135 (52.3%)	16 (69.6%)	0.28
Combination of AR	254 (90.4%)	236 (91.5%)	18 (78.3%)	0.06
Exposure to tobacco	137 (48.8%)	125 (48.4%)	12 (52.2%)	0.83
Receiving ICS	200 (71.2%)	185 (71.7%)	15 (65.2%)	0.48
tIgE (kU/L)	455.0 (195.7, 865.5)	473.5 (201.5, 924.3)	366.3 (134.8, 479.1)	0.01
*D. pteronyssinus*-sIgE (kU_A_/L)	67.8 (36.5, 105.0)	68.1 (34.8, 106.3)	52.7 (45.7, 70.6)	0.44
*D. farinae*-sIgE (kU_A_/L)	58.4 (33.3, 95.1)	60.6 (31.4, 98.5)	44.0 (35.4, 61.9)	0.24
BEC (cells/μL)	493 (320, 745)	505 (328, 761)	410 (260, 590)	0.23
FeNO (ppb)	29.3 (15.0, 55.5)	28.5 (14.9, 56.4)	40.0 (28.0, 52.5)	0.30
FEV1%Pred (%)	94.8 (87.3, 102.6)	94.5 (87.1, 102.3)	99.8 (90.3, 109.2)	0.31
C-ACT	23 (21, 25)	23 (21, 25)	21 (20, 25)	0.16

AR: allergic rhinitis; BEC: blood eosinophil count; BMI: body mass index; C-ACT: Childhood Asthma Control Test; FeNO: fractional exhaled nitric oxide; FEV1%Pred: percentage of forced expiratory volume in 1 s to the predicted value; ICS: inhaled corticosteroids; tIgE: total IgE; sIgE: allergen-specific IgE

### Sensitization rates of fungi and *Staphylococcal* enterotoxins

**Fig. 3:**
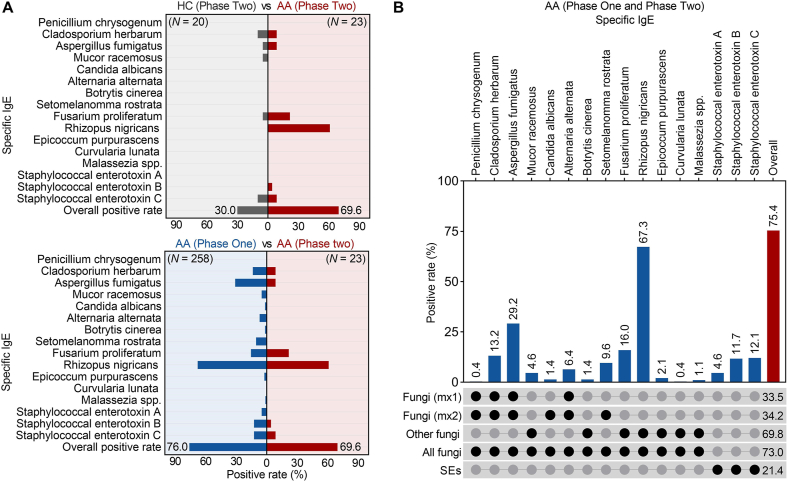
Positive rates of fungal sIgE and *Staphylococcal enterotoxin*-sIgE. (A) Comparison of positive rates: between AA and HC in Phase Two, and between AA in Phase One and Phase Two. (B) Positive rates in all AA children from both Phase One and Phase Two. AA: allergic asthma; HC: healthy control; sIgE: allergen-specific IgE; mx1: mixture of *Penicillium chrysogenum*, *Cladosporium herbarum*, *Aspergillus fumigatus*, *Alternaria alternata*; mx2: mixture of *Penicillium chrysogenum*, *Cladosporium herbarum*, *Aspergillus fumigatus*, *Candida albicans*, *Alternaria alternata*, *Setomelanomma rostrata*; SE: *Staphylococcal* enterotoxin

The sensitization rate of fungi and SEs in healthy children was 30.0% (6/20), which was significantly lower than the 69.6% (16/23) observed in AA children during Phase Two (*P* = 0.01). However, the sensitization rates in AA children during Phases One (76.0%, 196/258) and Two were not significantly different (Fig. 3A). The overall sensitization rate in AA children was 75.4% (212/281, Fig. 3B). The rates were comparable between those residing in Chongqing and those outside Chongqing (76.9% vs 73.6%, *P* = 0.52). The concentrations of individual sIgE are detailed in Appendix 9. RN exhibited the highest sensitization rate at 67.3% (189/281). Sensitization rates for the common fungal mixtures, mx1 and mx2, were 33.5% (94/281) and 34.2% (96/281), respectively. Among the 185 children who were not sensitized to mx2, 116 (62.7%) were sensitized to at least 1 other fungus or SE, compared to 10.3% when RN was not considered.

An assessment of the cross-reactivity between RN-sIgE and other sIgE suggested a low likelihood of false positives (Appendix 10). Furthermore, RN-sIgE levels were re-measured in 26 AA children using the Phadia system. While the results differed from those obtained with the HRTJ system, the correlation between the 2 systems was strong (*Rho* = 0.96, *P* < 0.001). This discrepancy was determined after a process of troubleshooting and exclusion, and the difference is likely due to variations in the raw materials used for the RN crude extract across the 2 systems (Appendix 11).

### Clinical associations of *Rhizopus nigricans* sensitization

**Fig. 4:**
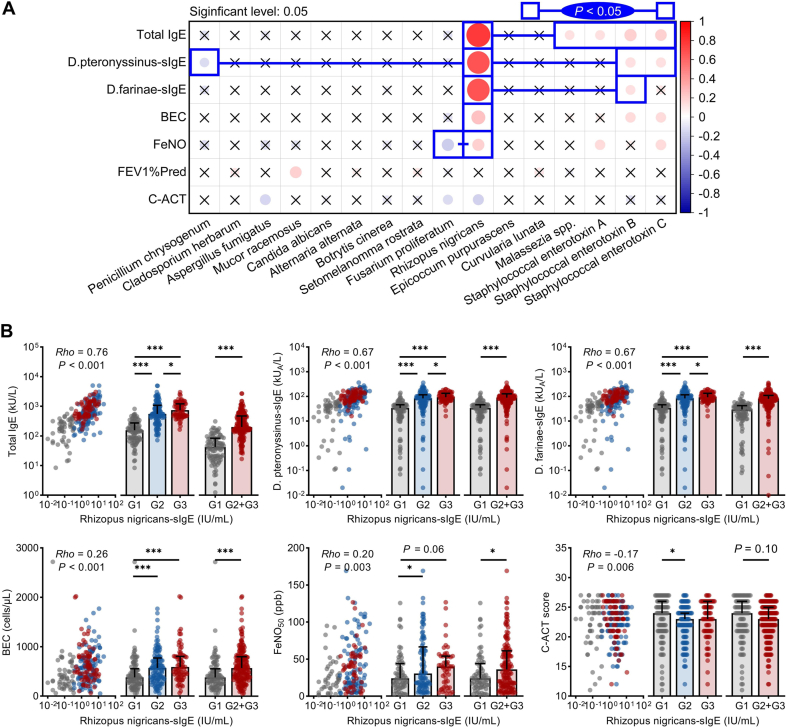
Clinical associations of *Rhizopus nigricans* sensitization. (A) Spearman correlation and coefficient comparisons among the fungal sIgE and SE-sIgE. (B) Stratified analysis of the clinical associations of RN-sIgE. C-ACT: Childhood Asthma Control Test; BEC: blood eosinophil count; FeNO: fractional exhaled nitric oxide; FEV1%Pred: percentage of forced expiratory volume in 1 s to the predicted value; sIgE: allergen-specific IgE; RN: *Rhizopus nigricans*; SE: *Staphylococcal enterotoxin*. G1: RN-sIgE negative group; G2: RN-sIgE and other sIgE positive group; G3: RN-sIgE positive alone group

Bivariate correlation analysis revealed that RN-sIgE and SE-sIgE were significantly associated with AA features, with RN-sIgE showing the highest coefficients (Fig. 4A). Multiple linear regression analysis revealed significant correlations between tIgE and *Aspergillus fumigatus*-sIgE, RN-sIgE, and SEB-sIgE, with RN-sIgE showing the strongest association (standardized *β* = 0.66). This suggests that among the analyzed fungal allergens, RN-sIgE has the most pronounced relative contribution to tIgE levels in the regression model (Appendix 12).

Patients were categorized into 3 groups on the basis of RN-sIgE levels: Group One included RN-sIgE negative children (G1, *N* = 92, 32.7%); Group Two included children positive for both RN-sIgE and other sIgE (G2, *N* = 115, 40.9%); and Group Three included children positive only for RN-sIgE (G3, *N* = 74, 26.3%). No significant differences were observed in the basic characteristics among the 3 groups (Appendix 13). Further analysis revealed that the level of RN-sIgE was positively correlated with tIgE, *D. pteronyssinus*-sIgE, *D. farinae*-sIgE, BEC, and FeNO and negatively correlated with C-ACT (Fig. 4B). Consistent trends and results were observed when RN-sIgE was analyzed as a categorical variable, which aligns with the sensitivity analysis results (Appendix 14). Additionally, no correlation was found between RN-sIgE and FEV1%Pred (*Rho* = −0.03, *P* = 0.64).

### Proteomic analysis of *Rhizopus nigricans* sensitization

**Fig. 5:**
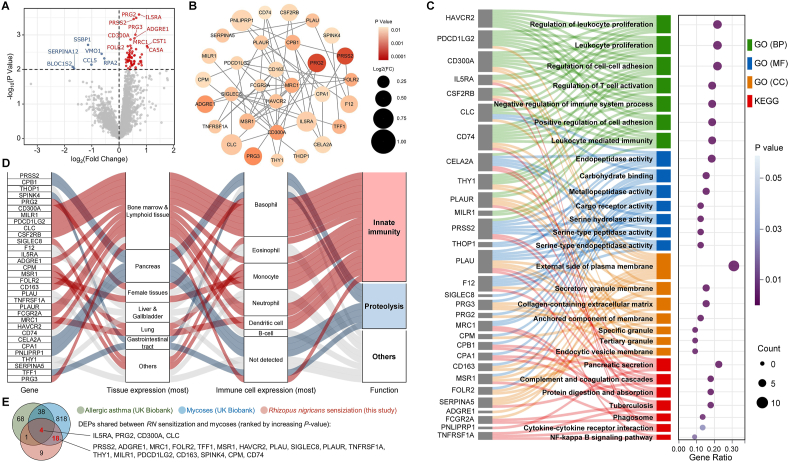
Proteomic analysis of *Rhizopus nigricans* sensitization. (A) Volcano plot of DEPs. (B) PPI network of DEPs. (C) GO and KEGG enrichment analysis of the PPI network. (D) Specific annotation of DEPs of interest. (E) Venn plot of shared DEPs between UK Biobank and this study. DEP: differentially expressed protein; GO: Gene Ontology; KEGG: Kyoto Encyclopedia of Genes and Genomes; PPI: protein-protein interaction; RN: *Rhizopus nigricans*

Olink proteomic analysis identified 67 DEPs, including 61 upregulated and 6 downregulated proteins (Fig. 5A). Further analysis revealed a PPI network consisting of 32 upregulated DEPs, with IL5RA and PRG2 showing the most significant levels, potentially reflecting an association with eosinophilic inflammation (Fig. 5B).

Functional enrichment analyses showed that these DEPs were significantly associated with biological processes such as leukocyte proliferation and adhesion, as well as molecular functions related to enzyme activity and proteolysis (Fig. 5C). KEGG pathway analysis further indicated that these DEPs a connection to the pancreatic secretion pathway. Specific annotation using the Human Protein Atlas database indicated that these DEPs were predominantly linked to innate immunity (53.1%) and proteolysis (21.9%, Fig. 5D). Additionally, among these 32 DEPs, 18 were shared with mycoses but not with AA in the UK Biobank, among which PRSS2 was the most significant (Fig. 5E). These findings suggest that *RN* sensitization has distinct molecular features.

### Statistical utility of tIgE in identifying *Rhizopus nigricans* sensitization

**Fig. 6:**
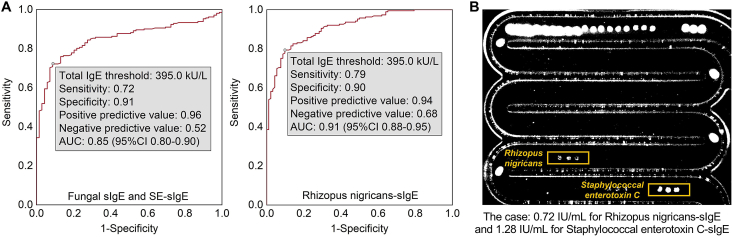
Statistical utility of tIgE in identifying fungal sIgE and *Staphylococcal enterotoxin*-sIgE. (A) ROC analysis of fungal sIgE and SE-sIgE through tIgE. (B) A typical clinical case of an asthmatic child with RN and SE sensitization. AUC: area under the curve; ROC: receiver operating characteristic; sIgE: allergen-specific IgE; tIgE: total IgE; RN: *Rhizopus nigricans*; SE: *Staphylococcal enterotoxin*

Multiple linear regression analysis revealed that sensitization to fungi was significantly associated with tIgE levels (*β* = 619.6, 95% CI 313.8–925.3 kU/L, *P* < 0.001). Given the potential statistical association between elevated tIgE levels and allergic sensitization,^24^, ^25^, ^26^, ^27^ further ROC analysis was conducted to evaluate the performance of tIgE as aa statistical indicator of fungal sensitization. The analysis suggested that a tIgE threshold of 395.0 kU/L could serve as an effective screening tool for identifying individuals at risk of fungal sensitization (AUC 0.85), including RN sensitization (AUC 0.91). This threshold may be particularly useful in clinical settings with pediatric asthma populations similar to our study where fungal sensitization is not routinely tested (Fig. 6A).

In a subsequent clinical case, a child with refractory asthma, previously categorized as “nonallergic,” was found to have a tIgE level of 461.4 IU/mL but negative results for 19 common sIgE tests. Further testing revealed fungal sIgE (RN-sIgE 0.72 IU/mL and SEC-sIgE 1.28 IU/mL) (Fig. 6B). A questionnaire survey further confirmed household fungal exposure (Appendix 15).

## Discussion

### Characteristics of fungal sensitization

This study broadens the spectrum of fungal and SE allergens identified in AA children from Southwest China. A key finding is the high sensitization rate of RN (67.3%), a fungus from the *Mucorales* order that thrives in humid environments and is commonly found in human living spaces.^28^ RN is known to cause crop and food spoilage and is frequently found in spoiled grains,^29^ bread,^30^ and approximately 3% of tap water samples.^31^ In Southwest China, persistent damp and moldy conditions may exacerbate food contamination by RN. Additionally, *Rhizopus* strains are widely used in liquor fermentation in this region,^32^ underscoring its pervasive environmental presence.

The sensitization rate of RN in this study is markedly higher than the 9.7% reported in asthmatic adults from Germany.^8^ This discrepancy may result from regional variations, differences in asthma phenotypes between adults and children, and varying allergen exposure levels.^33^ Notably, the sensitization rate of *Rhizopus* in Germany surged by 57.2% from 1998 to 2007 to 2008–2017,^7^ highlighting a potential temporal trend that merits further exploration. Similarly, an Indian study revealed that despite its relatively low airborne spore concentration, RN remains prevalent year-round. Moreover, RN exhibits a high sensitization rate (41.3%) in patients with respiratory allergies, with the most severe sensitization observed in this group.^34^ Environmental RN concentrations have also been closely linked to respiratory damage,^35^ and significantly elevated tIgE levels.^36^ In agreement with these findings, our study demonstrated that among all measured fungal and SE-sIgE, RN-sIgE exhibited the strongest associations with clinical features and elevated tIgE levels.

Based on the statistics of reported Rhizopus distributions in China, it has been found that *Rhizopus* species are widely present in soil and humus samples across various provinces.^37^ However, outdoor air monitoring in Chengdu did not identify *Rhizopus* as a dominant fungus.^38^ Additionally, DNA sequencing of household fungi in Chongqing did not identify *Rhizopus* among the top 15 detected genera or species.^4^ The most likely explanation is that indoor exposure to Rhizopus primarily occurs through food sources, such as fruits in the kitchen and refrigerator,^39^^,^^40^ which are not typically recommended sites for household dust collection for allergen detection according to the Chinese expert consensus.^41^ These factors collectively contribute to the low DNA concentrations of RN in household dust samples. This hypothesis may also explain the observed upregulation of innate immunity and pancreatic proteolytic enzyme secretion in AA children sensitized to RN, possibly as a defensive response to inactivate the fungus or its bioactive products in the digestive tract. Notably, 2 key DEPs, PRG2 and PRSS2, function as cytotoxins and defensins in the blood and digestive tract. This raises an intriguing question: could fungal exposure via the digestive tract significantly influence pulmonary immunity in children with AA? This concept aligns with the gut-lung axis proposed in recent studies.^42^ Therefore, future household dust collection for allergen detection may need to consider areas such as food surfaces and places where food is stored.

Another potential explanation is that *Rhizopus* products are known for their immune-enhancing properties,^43^ which could have significant effects on the body even at relatively low concentrations, as shown in the Indian study.^34^ *Rhizopus* is widely used in industrial applications due to its diverse and highly active enzymes, such as lipases used in detergents.^44^ The epithelial barrier hypothesis suggests that inhaling enzymes could contribute to asthma,^45^ potentially explaining why aeroallergens often exhibit protease activity.^46^ Overall, the mechanisms underlying the marked increase in type 2 immunity caused by RN remain unclear.

### Clinical relevance of fungal sensitization

Currently, 121 fungal and bacterial allergens are officially recognized by the WHO/IUIS Allergen Nomenclature Sub-Committee (allergen.org). Given the sample volume requirements and costs associated with each test,^47^ comprehensive singleplex detection of multiple fungal sIgE antibodies remains impractical, particularly in pediatric populations. Therefore, defining a targeted allergen panel tailored to regional and environmental factors is crucial. In this context, this study explored the potential of microfluidic chip technology, which offers advantages in automation and scalability. The combined detection (mx2) of *Penicillium chrysogenum*, *Cladosporium herbarum*, *Aspergillus fumigatus*, *Candida albicans*, *Alternaria alternata*, and *Setomelanomma rostrata* yielded a positive rate of 34.2%, comparable to previous reports.^6^^,^^18^ Notably, RN emerged as the most prevalent “hidden” allergen among AA children who were not sensitized to mx2. These findings suggest that RN should be considered as an additional detection target in clinical practice.

Previous studies have indicated that tIgE levels can serve as a statistical marker for allergic sensitization,^24^, ^25^, ^26^, ^27^ with fungal sIgE exhibiting stronger correlations with tIgE than other common allergens, particularly in AA children.^33^ Building on these findings, our study evaluated the utility of tIgE in identifying fungal sensitization, demonstrating that tIgE exhibited strong predictive power for RN sensitization (AUC 0.91). Furthermore, we presented a clinical case of unrecognized fungal sensitization, underscoring the potential of tIgE as a screening tool for fungal allergens. These results provide practical insights into the interpretation of markedly elevated tIgE levels in AA children, suggesting that the risk of fungal sensitization should be carefully considered in clinical assessments.

AA children sensitized to fungi, may represent distinct endotype, as fungal allergens are known to strongly activate the innate immune response and can simultaneously promote the polarization of Th17 cells.^48^ Traditionally, Th17 responses have been thought to be more prevalent in Th2-low asthma patients,^49^ typically associated with severe asthma and poor treatment responsiveness,^50^ which is consistent with the asthma phenotype induced by fungal sensitization.^2^ However, an increasing number of studies have shown that fungi can induce the polarization of both Th2 and Th17 cells,^51^ as well as the capacity for Th17 to Th2 transition.^52^ Moreover, anti-IgE therapy has also demonstrated efficacy in treating severe asthma associated with fungal sensitization.^53^ Notably, in our proteomic analysis of RN-sensitized children, we did not observe significant upregulation of the IL-17 or IL-4/IL-13 pathways. These findings highlight the complex role of fungi in asthma immune responses.

Our proteomic analysis revealed that among DEPs upregulated in RN-sensitized children, more overlapped with mycoses-associated proteins in the UK Biobank than with AA-associated proteins. However, despite the growing recognition of fungal allergens, targeted treatment strategies remain limited. Allergen immunotherapy for *Alternaria alternata* has demonstrated initial efficacy,^54^ but challenges in standardizing *Alternaria* extracts have hindered its widespread adoption due to concerns regarding efficacy and safety.^55^ Notably, our study identified IL5RA as the most significantly upregulated DEP in RN-sensitized AA children, suggesting that anti-IL5RA therapy (benralizumab) may benefit these patients, as supported by previous case reports.^56^ Additionally, in patients with allergic bronchopulmonary aspergillosis, anti-eosinophil treatment is generally effective,^57^ but there are exceptions (eg, switching from benralizumab to dupilumab for efficacy).^58^ This is consistent with our Olink results, which show that although the most prominent DEP is eosinophil-related, there are still many other DEPs, which may account for the heterogeneity in treatment responses. Further research is warranted to refine both diagnostic and therapeutic strategies for fungal sensitization in clinical practice.

### Technical challenges in fungal sIgE detection

The detection of fungal sIgE remains technically challenging, primarily due to the generally low serum concentrations of fungal sIgE, necessitating improvements in assay sensitivity. One promising approach involves the use of nanobodies as secondary antibodies in sIgE detection systems,^59^ which may enhance sensitivity and specificity. However, while highly sensitive detection methods improve assay performance, they may also increase the likelihood of false positives. This issue is particularly relevant when crude allergen extracts are used for multiplex sIgE detection, as cross-reactivity between allergenic components can compromise result interpretation.^60^

Another challenge concerns the clinical threshold for defining positive sIgE results. The conventional cutoff of 0.35 kU_A_/L may not be optimal for fungal allergens, and recent evidence suggests that a lower threshold, such as 0.1 kU_A_/L, could also be reasonable.^61^ Further investigation is needed to determine the most appropriate threshold for fungal sensitization. Additionally, the lack of standardized fungal allergen preparation remains a significant limitation.^13^ Variability in fungal strains due to differences in culture conditions, developmental stages, and extraction protocols contributes to inconsistencies in allergen composition and concentration. In our study, discrepancies in RN crude extracts from different manufacturers resulted in differences between the HRTJ and Phadia systems, underscoring the need for standardization to ensure comparability across studies and clinical settings.

### Limitations

This study provides valuable insights into fungal sensitization in AA children while also highlighting areas for further investigation. First, the disparity in group sizes between AA children and healthy controls may introduce potential bias, as smaller control groups can reduce statistical power and affect the robustness of comparative analyses. Second, our study was limited to AA children from Southwest China, a region known for its humid climate. Therefore, the findings may not be generalizable to populations in other geographic regions with different climatic characteristics. Third, the absence of functional validation for RN-sIgE, combined with observed discrepancies in RN-sIgE detection between the Phadia and HRTJ systems, underscores the need for further validation studies to clarify the allergenic components of RN and their clinical significance. Finally, the sample size calculation was based on the overall sensitization rate, which may partially weaken the confidence in intergroup analyses.

## Conclusion

In summary, this study demonstrates that fungal sensitization is common among AA children in Southwest China, with RN emerging as a potentially underestimated fungal allergen associated with asthma-related clinical features. Sensitization to RN may be linked to the upregulation of innate immune responses and the secretion of pancreatic proteolytic enzymes. Moving forward, fungal sensitization should be explored from fundamental, clinical, technological, and translational perspectives to further elucidate its role in asthma pathogenesis and improve diagnostic and therapeutic strategies.

## Abbreviations

(AUC), area under curve; (BEC), blood eosinophil count; (C-ACT), Childhood Asthma Control Test; (DEP), differential protein expression; (FeNO), fractional exhaled nitric oxide; (FEV1%Pred), forced expiratory volume in 1 s to the predicted value ratio; (GO), Gene Ontology; (ICSs), inhaled corticosteroids; (KEGG), Kyoto Encyclopedia of Genes and Genomes; (PPI), protein-protein interaction; (*RN*), *Rhizopus nigricans*; (ROC), receiver operating characteristic; (*SEs*), *Staphylococcal* enterotoxins; (sIgE), allergen-specific IgE; (tIgE), total IgE.

## Data sharing statement

The data that support the findings of this study are available from the corresponding author upon reasonable request.

## Authors’ contributions

HF: Conceptualization, Methodology, Software, Formal analysis, Investigation, Resources, Data curation, Writing - original draft, Visualization. JL and XL: Methodology, Software, Formal analysis, Writing - original draft, Visualization. XW, DZ, YT and RW: Investigation, Data curation, Writing - original draft. NZ and WZ: Resources, Writing - review & editing, Supervision, Funding acquisition. LR and EL: Conceptualization, Methodology, Validation, Writing - review & editing, Project administration, Funding acquisition.

## Ethics approval and informed consent

This study was approved by the Ethics Committee of Children's Hospital of Chongqing Medical University (Nos. 2018-2, 2019-27-1, 2023-102, 2023-103). Written informed consent was obtained from the legal guardians of all children with allergic asthma.

## Authors’ consent for publication

The authors confirmed that neither the entire paper nor its content has been submitted, accepted, or published elsewhere. All authors have approved the manuscript and agree with the submission.

## Funding

This study was supported by the Major Program of National Clinical Research Center for Child Health and Disorders in Children's Hospital of Chongqing Medical University (Grant No. NCRCCHD-2023-MP-01) and the Science and Technology Research Program of Chongqing Municipal Education Commission (Grant No. KJZD-M202200404).

## Declaration of competing interest

The authors declare that they have no conflicts of interest.
