# Supplementary material for: High sensitization to Rhizopus nigricans in children with allergic asthma in Southwest China: A microfluidic chip and proteomics study
Source: World Allergy Organ J. 2025 Aug 8;18(8):101097. doi: 10.1016/j.waojou.2025.101097 (PMC12355534; doi:10.1016/j.waojou.2025.101097)
Supplement: Multimedia component 1 [file mmc1.docx]

**High Sensitization to *Rhizopus nigricans* in Children with Allergic Asthma in Southwest China: A Microfluidic Chip and Proteomics Study**

**Appendices**

**Contents**

[Appendix 1. Evaluation of the Childhood Asthma Control Test. 2](#_Toc186367801)

[Appendix 2. Target allergens (species), supporting literature and companies. 2](#_Toc186367802)

[Appendix 3. Development and verification of the microfluidic chip. 3](#_Toc186367803)

[Appendix 4. Sample detection procedure using the microfluidic chips. 6](#_Toc186367804)

[Appendix 5. General information and clinical characteristics of AA children in Olink proteomic analysis. 7](#_Toc186367805)

[Appendix 6. The process and data generation of Olink proteomics. 7](#_Toc186367806)

[Appendix 7. Sample size and confidence level calculation. 7](#_Toc186367807)

[Appendix 8. Processing of the missing data. 8](#_Toc186367808)

[Appendix 9. The concentration of each fungal sIgE and SE-sIgE (HRTJ system, IU/mL). 9](#_Toc186367809)

[Appendix 10. Evaluation of the cross-reactivity of RN-sIgE. 10](#_Toc186367810)

[Appendix 11. Comparison of the RN-sIgE levels between HRTJ and Phadia systems 11](#_Toc186367811)

[Appendix 12. Multiple linear regression of sIgE to tIgE. 14](#_Toc186367812)

[Appendix 13. Grouping strategy based on RN-sIgE and comparisons of basic characteristics. 15](#_Toc186367813)

[Appendix 14. The sensitivity analysis for stratified analysis of RN-sIgE. 15](#_Toc186367814)

[Appendix 15. The details of the case. 16](#_Toc186367815)

**Appendix 1. Evaluation of the Childhood Asthma Control Test.**

The Asthma Control Test (ACT) is a five-question questionnaire designed to assess asthma control, with a maximum score of 25 points.^1^ The ACT is intended for asthma patients aged 12 and older, as it was designed and validated for this age group. Liu et al.^2^ developed the Childhood Asthma Control Test (C-ACT) to evaluate asthma control in children aged 4 to 11 years. The C-ACT consists of seven questions, with a total score of 27, and has shown good clinical relevance in assessing asthma control in Chinese children.^3^ Combining the ACT and C-ACT for analysis may introduce systematic errors due to their differing questions and scoring systems. To ensure consistent evaluation and rational statistical analysis, all children in this study were assessed using the C-ACT. Although the C-ACT has not been specifically validated for adolescents aged 12 and above, this expanded age range has produced recognized research results both domestically and internationally.^4,5^ This study evaluated the correlation between age and C-ACT scores to determine whether age influences C-ACT outcomes. The results showed no correlation between C-ACT scores and age (*Rho* = -0.02, *P* = 0.76), and no significant difference in scores between the age groups (*P* = 0.88). Additionally, there was no significant difference in the proportion of asthma control between the age groups (*P* = 0.17).


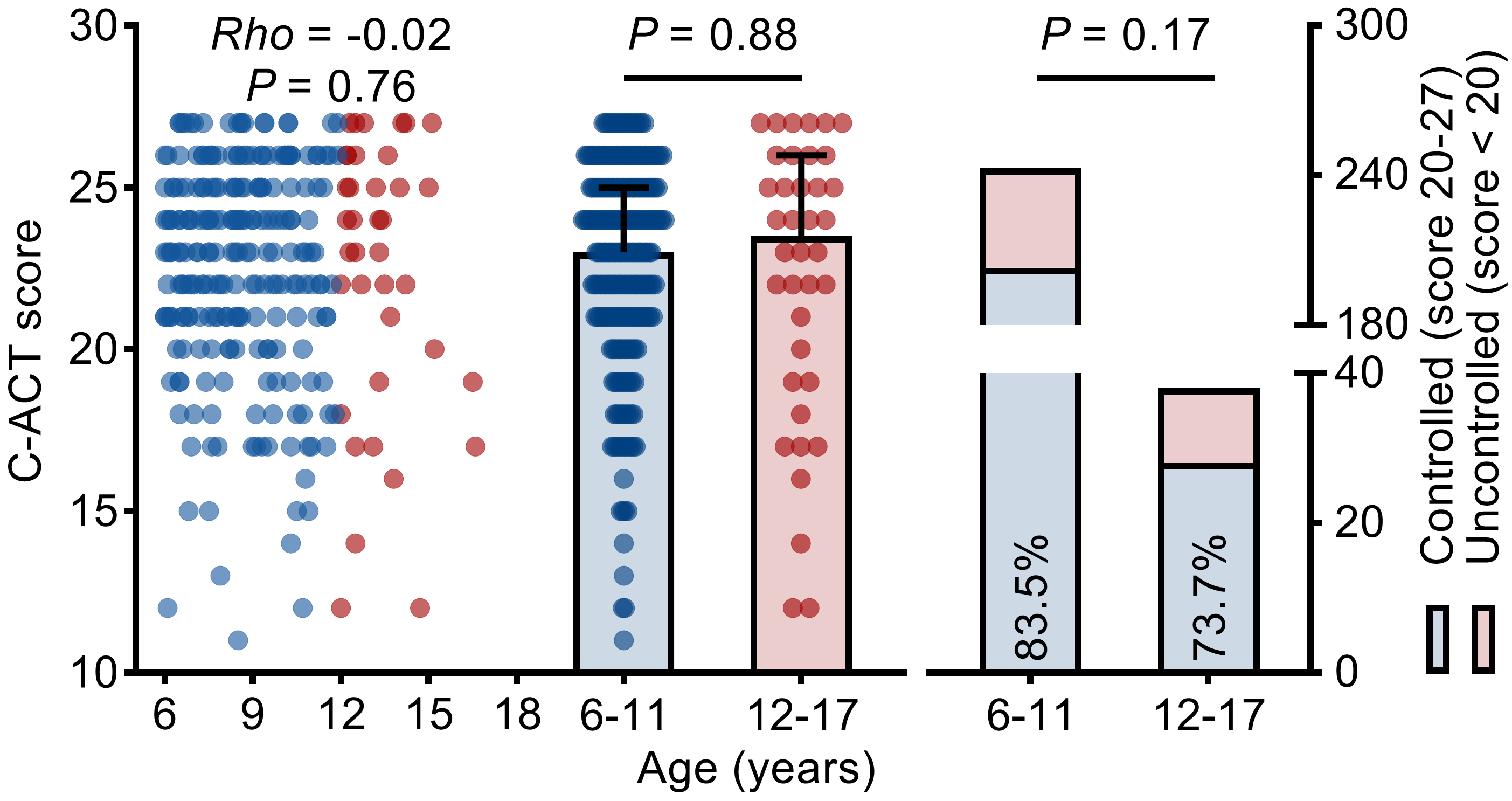


**References:**

1. Nathan RA, et al. Development of the asthma control test: a survey for assessing asthma control. J Allergy Clin Immunol. 2004.
2. Liu AH, et al. Development and cross-sectional validation of the Childhood Asthma Control Test. J Allergy Clin Immunol. 2007.
3. Yang Y, et al. Clinical application of childhood asthma control test [in Chinese]. Chinese Journal of Applied Clinical Pediatrics. 2017.
4. Rao H, et al. The application of childhood asthma control test (C-ACT) in children with asthma [in Chinese]. Journal of Chinese Physician. 2009.
5. Carroll WD, et al. Parent misperception of control in childhood/adolescent asthma: the Room to Breathe survey. Eur Respir J. 2012.

**Appendix 2. Target allergens (species), supporting literature and companies.**

| Name of the species | Code | PMID | Company | Address |
| --- | --- | --- | --- | --- |
| *Penicillium chrysogenum* | m1 | 25159468 | Rellergen Biotechnology | China Medical City Phase 5 Building G, Taizhou, China |
| *Fusarium proliferatum* | m9 | 28335387 |  |  |
| *Aspergillus fumigatus* | m3 | 35205913 | Diagnostische Systeme & Technologien GmbH | Güterbahnhofstrasse 16, 19059 Schwerin, Germany |
| *Mucor racemosus* | m4 | 34606833 |  |  |
| *Botrytis cinerea* | m7 | 20047250 |  |  |
| *Setomelanomma rostrata* | m8 | 17304882 |  |  |
| *Rhizopus nigricans* | m11 | 33292478 |  |  |
| *Epicoccum purpurascens* | m14 | 19239492 |  |  |
| *Curvularia lunata* | m16 | 34258653 |  |  |
| *Malassezia spp.* | m227 | 18834647 |  |  |
| *Cladosporium herbarum* | m2 | 22284927 | Stallergenes Greer | 639 Nuway Cir, Lenoir, NC 28645, USA |
| *Candida albicans* | m5 | 19689458 |  |  |
| *Alternaria alternata* | m6 | 34682259 | Medical & Biological Laboratories | 2-11-8 Shibadaimon, Minato-ku, Tokyo, Japan |
| *Staphylococcal enterotoxin A* | m80 | 31980492 | Toxin Technology | 7165 Curtiss Ave, Sarasota, FL, USA |
| *Staphylococcal enterotoxin B* | m81 | 31980492 |  |  |
| *Staphylococcal enterotoxin C* | m223 | 31980492 |  |  |

The allergen extracts are accompanied by Certificates of Analysis (COA) to assess product quality. However, since the extracts are crude allergen extracts, the purity of the extracts cannot be directly tested. The COA reports provide detailed information on various quality parameters, including the description of the product’s characteristics and compliance with specified standards.

**Appendix 3. Development and verification of the microfluidic chip.**

The HRTJ system used in our study is an optimized version of the BioIC system. The BioIC system has been used for IgE testing in clinical and research settings since around 2010. Studies have specifically compared the BioIC system with the Phadia (ImmunoCAP) or other systems. The references for these studies are as follows:

1. Shyur SD, et al. Determination of multiple allergen-specific IgE by microfluidic immunoassay cartridge in clinical settings. Pediatr Allergy Immunol. 2010.
2. Huang Z, et al. Application of Biochip Microfluidic Technology to Detect Serum Allergen-specific Immunoglobulin E (sIgE). J Vis Exp. 2019.
3. Szymczak-Pajor I, et al. Comparison of an automated microfluidic immunoassay technology (BioIC, lab-on-chips) and ImmunoCAP assay. Lab-on-chips as a tool for specific IgE (sIgE) detection. Postepy Dermatol Alergol. 2020.
4. Liang ZY, et al. A quantum dot-based lateral flow immunoassay for the rapid, quantitative, and sensitive detection of specific IgE for mite allergens in sera from patients with allergic rhinitis. Anal Bioanal Chem. 2020.

Additionally, other studies have utilized the BioIC system for sIgE detection in various fields of research, as well as for detecting other proteins. The references for these studies are as follows:

1. Yong SB, et al. Different profiles of allergen sensitization in different ages and geographic areas in Changhua, Taiwan. J Microbiol Immunol Infect. 2013.
2. Yao TC, et al. Multiplexed immunoglobulin E sensitization in relation to exhaled nitric oxide in a population sample of children. Allergy. 2014.
3. Liu PJ, et al. Specific Immunoglobulin G4 and Immunoglobulin E Titers to Common Food Antigens in Sera of Children With Allergic Rhinitis. Altern Ther Health Med. 2018.
4. Lin CH, et al. The Mediating Effect of Cytokines on the Association between Fungal Sensitization and Poor Clinical Outcome in Asthma. Biomedicines. 2022.
5. Chen CS, et al. Monitoring algorithm of hospitalized patients in a medical center with SARS-CoV-2 (Omicron variant) infection: clinical epidemiological surveillance and immunological assessment. PeerJ. 2023.

This study utilized microfluidic technology developed by HRTJ (BioIC) to construct the microfluidic chip, with crude allergen extracts immobilized onto blank chips. The chip design incorporated several key features:**(1)** internal calibration points, positioning points (positive control), and background points (negative control) for quality control, with each chip generating its own internal calibration curve and performing its own positive and negative controls; **(2)** systematic arrangement of detection items with adequate spacing to minimize interference; **(3)** three technical repetitions per test item, with the average reading recorded as the final result; **(4)** a designated tIgE site for comparison with Phadia system results to assess reliability, strategically positioned at the liquid flow outlet to mitigate potential substrate detachment effects on downstream sites caused by high tIgE levels; and **(5)** detection spots with a diameter of approximately 200 μm, spaced about 320 μm apart. Optical signal analysis between spots confirmed the absence of cross-contamination or interference between allergen spots.


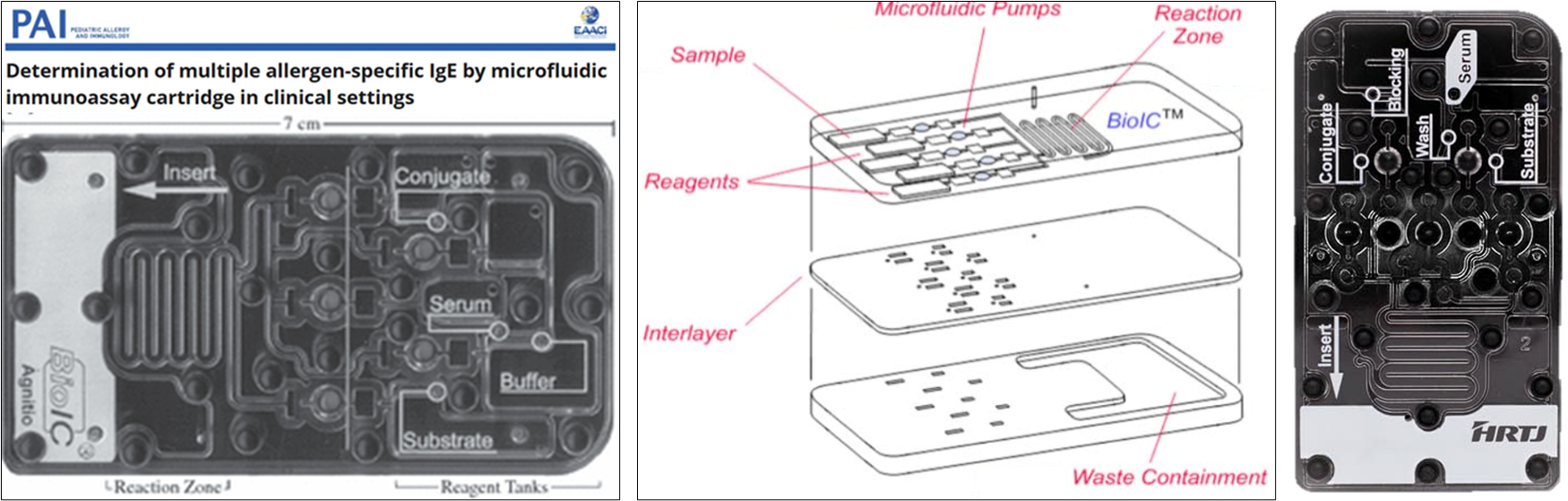


A total of 26 serum samples were collected to verify the chips, including 16 cases of fungal sensitization for positive verification and 10 healthy controls for negative verification. Positive verification criteria included individuals who tested positive for mx1 (a mixture of *Penicillium chrysogenum*, *Cladosporium herbarum*, *Aspergillus fumigatus*, and *Alternaria alternata*) or mx2 (a mixture of *Penicillium chrysogenum*, *Cladosporium herbarum*, *Aspergillus fumigatus*, *Candida albicans*, and *Alternaria alternata*) using the Phadia system. Notably, positive verification cases were not required to have a diagnosis of asthma. Negative verification included individuals without allergic symptoms or diseases, and negative mx1 or mx2 test results. The collected serum samples were analyzed using the HRTJ system, which clearly identified negative and positive groups.


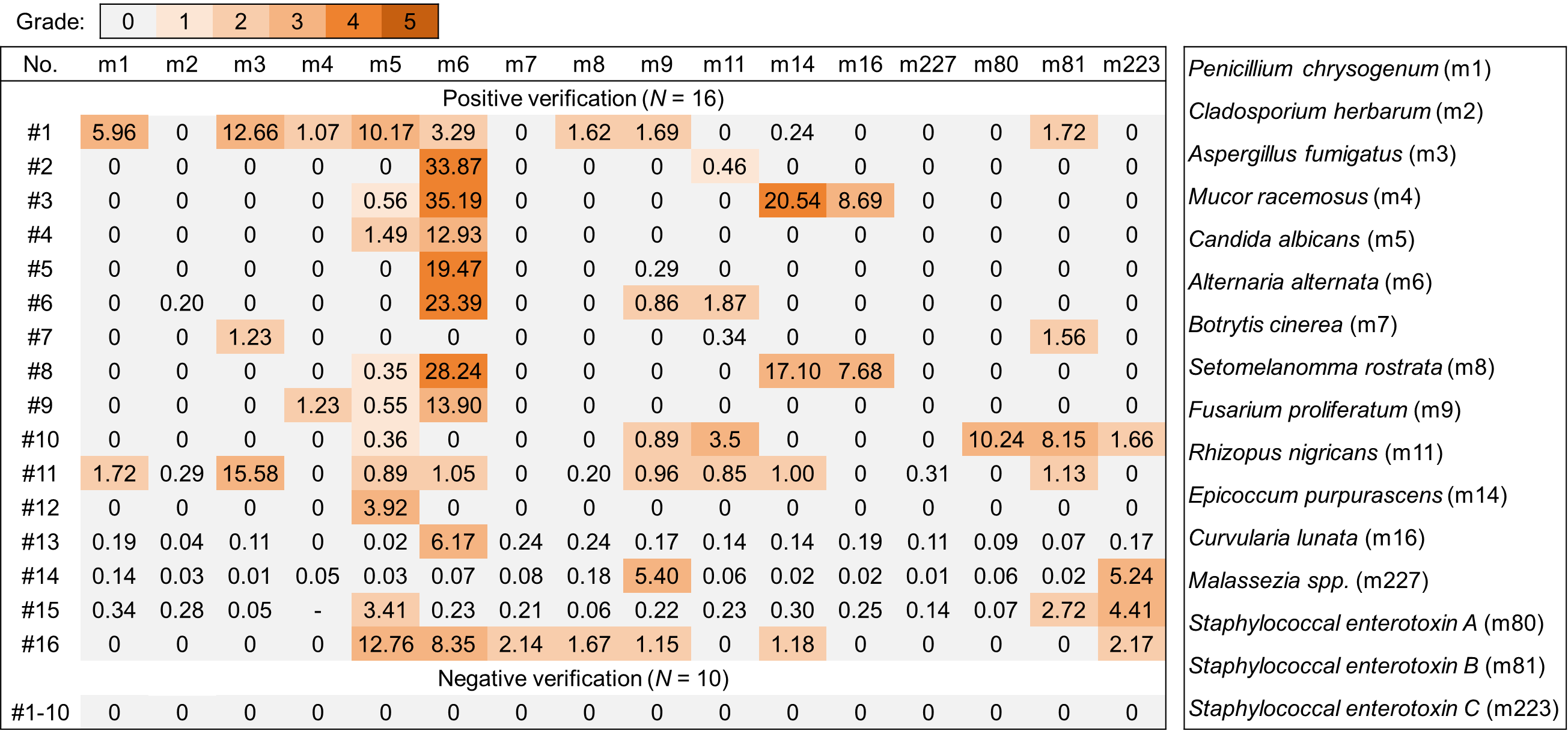


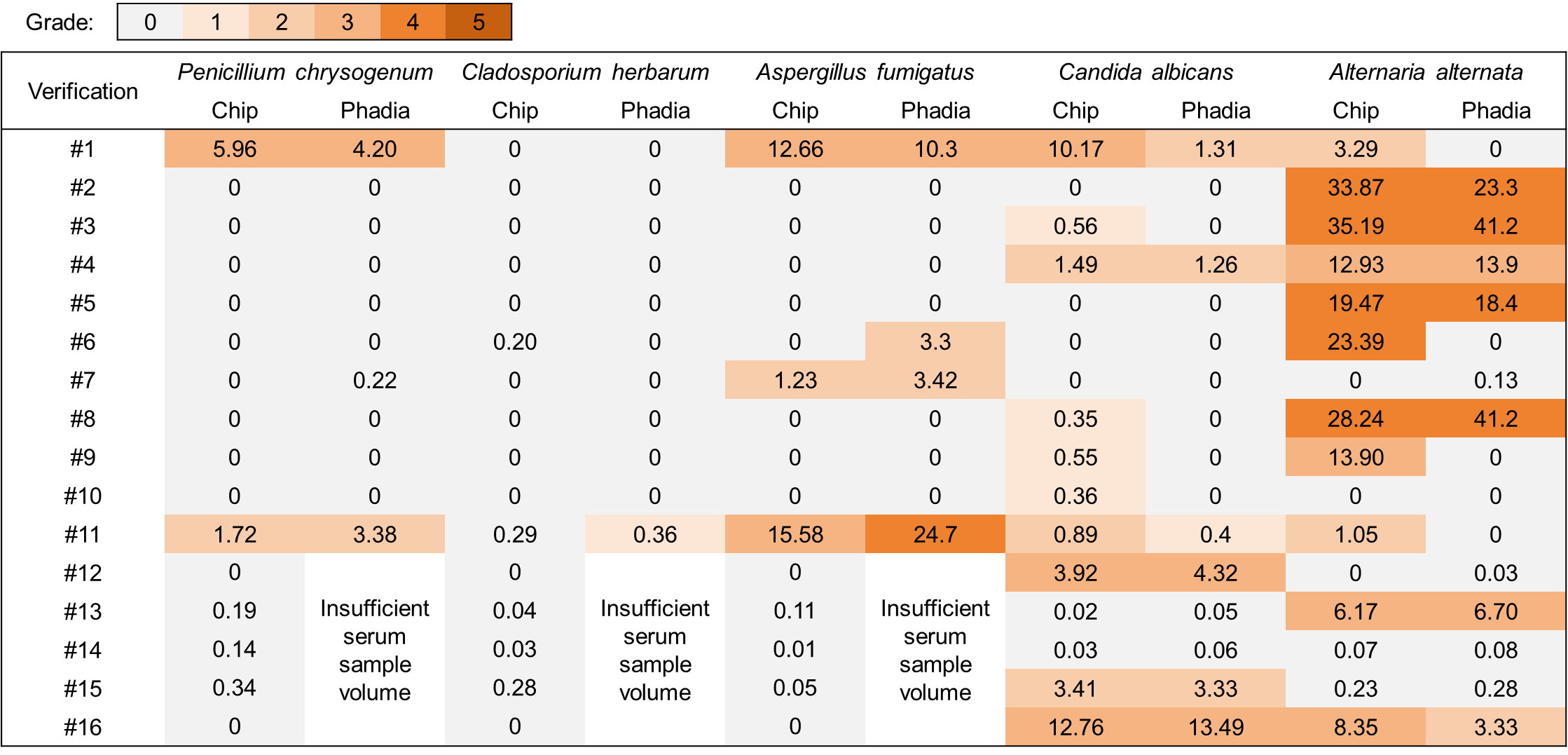


Further confirmation of sIgE levels in positive verification samples was conducted via the Phadia system, including *Penicillium chrysogenum*, *Cladosporium herbarum*, *Aspergillus fumigatus*, *Candida albicans*, and *Alternaria alternata*. The selection of these testing items was based on their registration and listing in China. The remaining items have yet to enter the sIgE testing market in China. Consequently, this study chose registered projects as representatives for result verification, comparing qualitative, semi-quantitative, and quantitative results. Comparison of the detection results between the two systems showed: **(1)** The qualitative results indicated 84.6% (55/65) consistency between the HRTJ and the Phadia system (sensitivity 0.90, specificity 0.82). **(2)** The semi-quantitative results showed 78.4% (51/65) consistency, with 92.3% (60/65) within plus or minus one level. **(3)** The quantitative results had a Spearman correlation coefficient of 0.72 (*P* < 0.001). These findings suggest that the microfluidic chip exhibits good consistency with the Phadia system.

**Appendix 4. Sample detection procedure using the microfluidic chips.**

All serum samples were tested from October 2023 to December 2023, using detection instruments such as a microfluidic chip (HRTJ), an MF1000 chemiluminescence analyzer (HRTJ), an MF100 incubator (HRTJ), a mini centrifuge (Sangon Biotech), a vortex oscillator (JoanLab), a 200 μL pipette (Eppendorf), a 250 μL pipette tip (Biosharp), a 1.5 mL centrifuge tube (Biosharp), and a test tube rack.

The detection process was as follows: **(1)** Thaw serum samples at 4°C in a refrigerator, then shake and mix, centrifuge immediately; **(2)** Prepare conjugate and substrate reagents according to the manufacturer’s instructions, shake well, and centrifuge; **(3)** Add 120 μL of conjugate reagent, 120 μL of blocking reagent, 620 μL of washing buffer, and 110 μL of serum sample to the corresponding wells; **(4)** Incubate samples for 35 minutes, followed by detection using the chemiluminescence analyzer. The reaction process was as follows: **(1)** The blocking reagent will seal the gaps in the reaction channels; **(2)** The sIgE in the serum samples will bind to the allergens spotted in the reaction channels; **(3)** The washing reagent will wash the reaction channels; **(4)** The enzyme-linked conjugate reagent will bind to the sIgE in the reaction channels; **(5)** The washing reagent will wash the channels again; **(6)** The chemiluminescence reagent will bind to the enzyme-conjugate complex, generating light intensity; **(7)** The low-temperature, high-sensitivity CCD will detect the light intensity at each allergen reaction point. For further details, the detection and reaction processes can be found at www.hongruitaijie.com/product.html.

The results were output as shown below after internal quality control and calibration were automatically performed by the program.


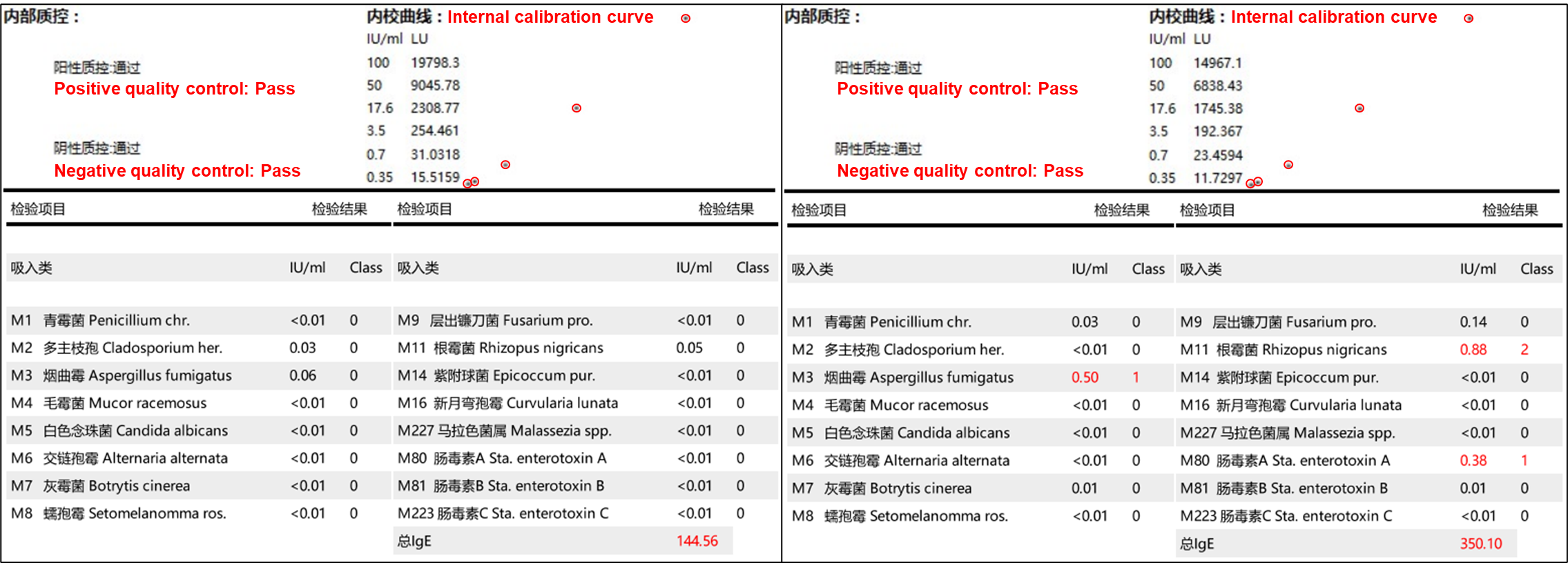


**Appendix 5. General information and clinical characteristics of AA children in Olink proteomic analysis.**

| Characteristics | Overall  (*N* = 46) | RN-sIgE negative  (*N* = 23) | RN-sIgE positive  (*N* = 23) | *P* value |
| --- | --- | --- | --- | --- |
| Age (year) | 9.8 (7.6, 11.9) | 8.6 (7.3, 11.9) | 10.3 (8.0, 12.2) | 0.46 |
| Sex, male | 30 (65.2%) | 15 (65.2%) | 15 (65.2%) | 1.00 |
| BMI-for-age Z score | 0.42 (-0.43, 1.08) | 0.18 (-0.42, 1.03) | 0.43 (-0.46, 1.22) | 0.70 |
| Region, Chongqing | 22 (47.8%) | 12 (52.2%) | 10 (43.5%) | 0.56 |
| Family history of allergies | 25 (54.3%) | 13 (56.5%) | 12 (52.2%) | 0.77 |
| Combination of AR | 38 (82.6%) | 19 (82.6%) | 19 (82.6%) | 1.00 |
| Exposure to tobacco | 18 (39.1%) | 9 (39.1%) | 9 (39.1%) | 1.00 |
| Receiving ICSs | 36 (78.3%) | 17 (73.9%) | 19 (82.6%) | 0.48 |
| tIgE (kU/L) | 720.0 (143.5, 1514.8) | 144.0 (90.4, 197.0) | 1481.0 (1239.0, 2210.0) | < 0.001 |
| *D. pteronyssinus*-sIgE (kU_A_/L) | 54.3 (25.3, 127.8) | 25.3 (14.9, 35.7) | 127.0 (93.6, 156.0) | < 0.001 |
| *D. farinae*-sIgE (kU_A_/L) | 59.4 (20.2, 109.3) | 20.8 (14.3, 33.9) | 109.0 (90.5, 145.0) | < 0.001 |
| BEC (cells/μL) | 440 (300, 800) | 340 (250, 532) | 590 (380, 1030) | 0.001 |
| FeNO (ppb) | 28.5 (17.3, 58.6) | 24.4 (15.2, 46.0) | 41.0 (20.3, 69.8) | 0.05 |
| FEV1%Pred (%) | 97.9 (89.3, 106.5) | 102.1 (94.6, 113.0) | 92.5 (87.0, 101.6) | 0.02 |
| C-ACT | 25 (21, 26) | 26 (24, 27) | 22 (20, 25) | 0.002 |
| AR: allergic rhinitis; BEC: blood eosinophil count; BMI: body mass index; C-ACT: Childhood Asthma Control Test; FeNO: fractional exhaled nitric oxide; FEV1%Pred: percentage of forced expiratory volume in one second to the predicted value; ICS: inhaled corticosteroids; tIgE: total IgE; sIgE: allergen-specific IgE; RN: *Rhizopus nigricans* | | | | |

**Appendix 6. The process and data generation of Olink proteomics.**

The process involves five core steps: **(1)** High-multiplex matched antibody pairs, labeled with unique DNA oligonucleotides, bind to their respective proteins in the samples. **(2)** Proximal oligonucleotides hybridize and are extended by DNA polymerase, forming a DNA barcode that is subsequently amplified by PCR. **(3)** Unique sample indexes are added to each sample, enabling pooling of DNA amplicons across all samples. This results in a separate library per panel. Each Olink library contains final DNA amplicons that include specific barcode sequences for each biomarker (or antibody pair), sample-specific indexes, and sequences necessary for Illumina sequencing (P5 and P7 Adapters, Sequencing Primer Binding Site Rd1SP). **(4)** Each Olink library is purified with Agentcourt AMPure XP magnetic beads, followed by quality assessment using a Bioanalyzer. **(5)** The Olink library is sequenced by NGS using Illumina® NextSeq™ 550, NextSeq™ 2000, or NovaSeq™ 6000.

The relative concentration of each biomarker is calculated using NPX Manager software. NPX, a relative protein quantification unit on a log_2_ scale, is derived from matched counts during the sequencing run. NPX data generation involves three key steps: normalization to the extension control (known standard), log_2_ transformation, and level adjustment using the plate control (plasma sample).

**Appendix 7. Sample size and confidence level calculation.**

The primary outcome measure was the rate of fungal and *SE* sensitization. Due to limited prior research, no reference data exists. This study determined the sample size based on the initial 20 tests. Results of fungal sIgE and SE-sIgE testing were categorized as “positive” or “negative”. Among the first 20 samples tested, 16 were positive for fungal sIgE and SE-sIgE, resulting in a positive rate of 80%, which was used as a reference for sample size calculation. The required sample size, calculated by PASS 15 software, was at least 264 cases. After completing sample detection, this study combined 258 individuals from Phase One with 23 individuals from the positive validation group in Phase Two, for a total sample size of 281. Among these, 212 were positive for fungal sIgE and SE-sIgE, resulting in a positive rate of 75.4%. Consequently, the confidence level achieved with the current sample size was 94%.


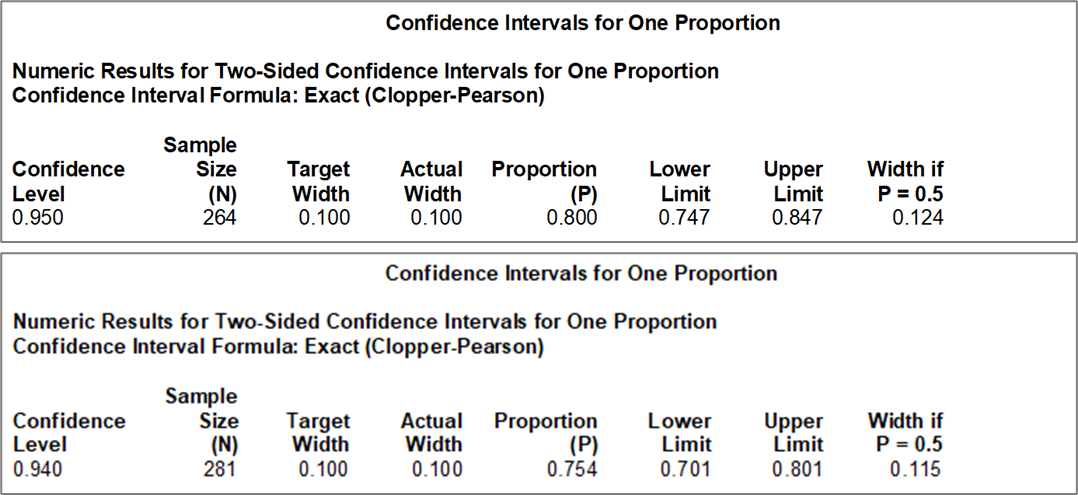


**Appendix 8. Processing of the missing data.**

This study used multiple imputation for variables with missing rates less than 20%. However, for FEV1% Pred and FeNO, which had missing rates above 20%, imputation was not performed because their deletions were linked to clinical assessments of asthma and were not completely random. Notably, the tIgE missing values (8.2% missing rate) were from positive validation samples in Phase Two. These samples were tested for tIgE using the HRTJ (BioIC) system, and the results filled the missing values without multiple imputation. Five datasets from multiple imputation were integrated into one new dataset by calculating the means for continuous variables and the modes for categorical variables. Statistical descriptions and correlation analyses were then performed on the original, interpolated, and new datasets to ensure consistency. Ultimately, the new dataset was used for formal analysis.

The general characteristics of the multiple imputation model

| Variables | Frequency | Missing rate | Role in the imputation model |
| --- | --- | --- | --- |
| Age | 281 | 0% | For prediction |
| Sex | 281 | 0% | For prediction |
| BMI-for-age Z score | 275 | 2.1% | For prediction and imputation |
| Region | 250 | 11.0% | For prediction and imputation |
| Family history of allergies | 274 | 2.5% | For prediction and imputation |
| Combination of AR | 281 | 0% | For prediction |
| Exposure to tobacco | 274 | 2.5% | For prediction and imputation |
| Receiving ICS | 281 | 0% | For prediction |
| Total IgE (Phadia system) | 258 | 8.2% | For prediction (replaced by HRTJ system) |
| *D. pteronyssinus*-sIgE | 258 | 8.2% | For prediction and imputation |
| *D. farinae*-sIgE | 258 | 8.2% | For prediction and imputation |
| BEC | 259 | 7.8% | For prediction and imputation |
| FeNO | 222 | 21.0% | For prediction |
| FEV1%Pred | 185 | 34.2% | For prediction |
| C-ACT score | 269 | 4.3% | For prediction and imputation |
| 16 fungal sIgE and SE-sIgE | 281 | 0% | For prediction |
| AR: allergic rhinitis; BEC: blood eosinophil count; BMI: body mass index; C-ACT: Childhood Asthma Control Test; FeNO: fractional exhaled nitric oxide; FEV1%Pred: percentage of forced expiratory volume in one second to the predicted value; ICS: inhaled corticosteroids; SE: *Staphylococcal enterotoxin* | | | |

Multiple imputation generated five datasets, resulting in seven datasets for comparison: the five imputed datasets, the raw dataset, and a new dataset. The results showed no significant differences among the imputed variables. Further analysis of fungal sIgE and SE-sIgE, RN-sIgE, and other variables indicated that the imputation did not alter the original dataset’s characteristics.

Basic characteristics of the imputation variables

| Variables, median (IQR) / n (%) | Dataset 1 | Dataset 2 | Dataset 3 | Dataset 4 | Dataset 5 |
| --- | --- | --- | --- | --- | --- |
| BMI-for-age Z score | 0.39 (1.86) | 0.39 (1.87) | 0.39 (1.89) | 0.39 (1.87) | 0.39 (1.87) |
| Region, Chongqing | 159 (56.6) | 157 (55.9) | 162 (57.7) | 160 (56.9) | 158 (56.2) |
| Family history of allergies | 151 (53.7) | 153 (54.4) | 150 (53.4) | 152 (54.1) | 152 (54.1) |
| Exposure to tobacco | 136 (48.4) | 137 (48.8) | 136 (48.4) | 135 (48.0) | 137 (48.8) |
| *D. pteronyssinus*-sIgE (kU_A_/L) | 67.8 (70.4) | 67.8 (71.4) | 67.3 (71.0) | 67.8 (69.7) | 67.8 (70.6) |
| *D. farinae*-sIgE (kU_A_/L) | 60.0 (68.0) | 60.0 (65.2) | 58.5 (67.8) | 58.5 (62.5) | 58.5 (65.7) |
| BEC (cells/μL) | 490 (459) | 480 (440) | 480 (435) | 482 (436) | 490 (450) |
| C-ACT score | 23 (4) | 23 (4) | 23 (4) | 23 (4) | 23 (4) |
| C-ACT: Childhood Asthma Control Test; BEC: blood eosinophil count | | | | | |


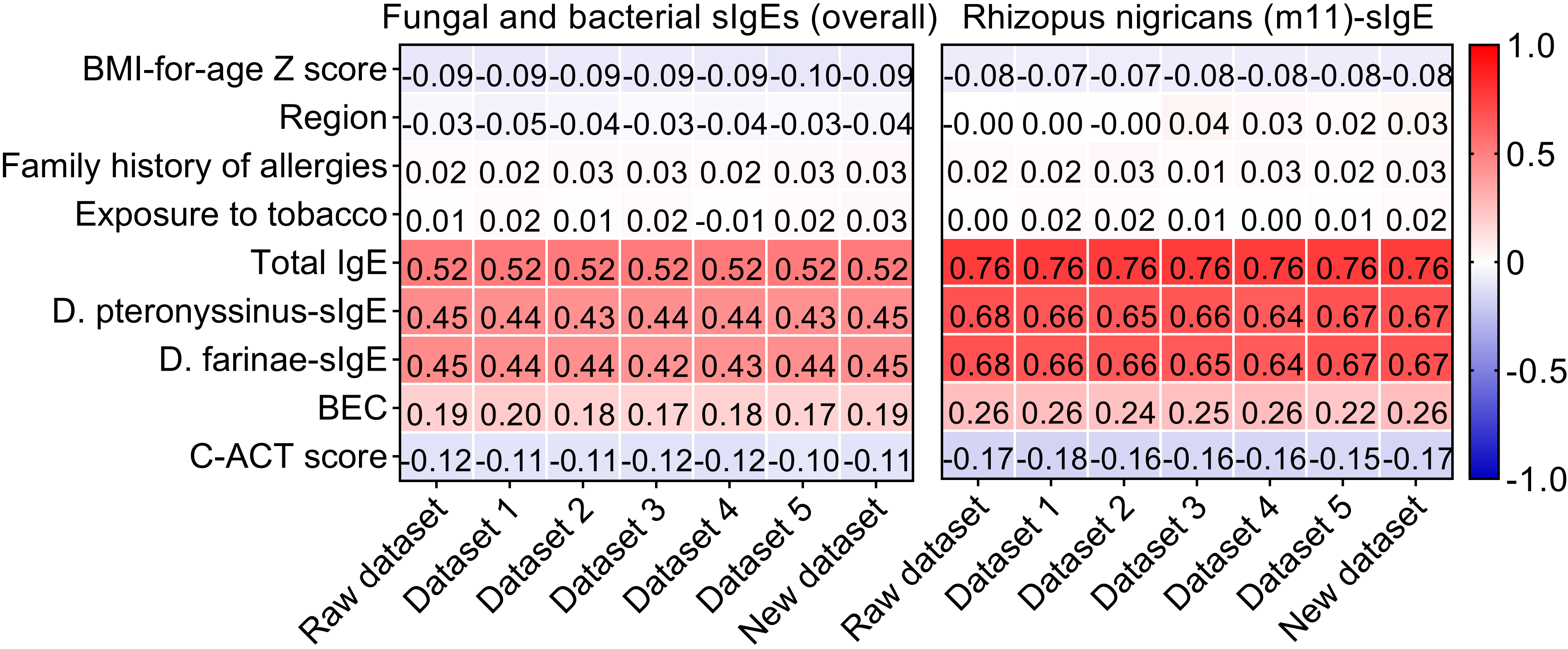


**Appendix 9. The concentration of each fungal sIgE and SE-sIgE (HRTJ system, IU/mL).**

| Allergen species | Min | P25^th^ | Median | P75^th^ | Max |
| --- | --- | --- | --- | --- | --- |
| *Penicillium chrysogenum* (m1) | 0 | 0.01 | 0 | 0.01 | 2.53 |
| *Cladosporium herbarum* (m2) | 0 | 0.13 | 0 | 0.13 | 1.68 |
| *Aspergillus fumigatus* (m3) | 0 | 0 | 0 | 0.58 | 3.26 |
| *Mucor racemosus* (m4) | 0 | 0 | 0 | 0.03 | 8.35 |
| *Candida albicans* (m5) | 0 | 0 | 0 | 0 | 0.73 |
| *Alternaria alternata* (m6) | 0 | 0 | 0 | 0 | 100.57 |
| *Botrytis cinerea* (m7) | 0 | 0 | 0 | 0 | 1.20 |
| *Setomelanomma rostrata* (m8) | 0 | 0 | 0 | 0 | 1.47 |
| *Fusarium proliferatum* (m9) | 0 | 0 | 0.04 | 0.21 | 5.91 |
| *Rhizopus nigricans* (m11) | 0 | 0.13 | 1.26 | 3.08 | 20.49 |
| *Epicoccum purpurascens* (m14) | 0 | 0 | 0 | 0 | 10.51 |
| *Curvularia lunata* (m16) | 0 | 0 | 0 | 0 | 1.75 |
| *Malassezia spp.* (m227) | 0 | 0 | 0 | 0 | 2.28 |
| *Staphylococcal enterotoxin A* (m80) | 0 | 0 | 0 | 0 | 19.07 |
| *Staphylococcal enterotoxin B* (m81) | 0 | 0 | 0 | 0.02 | 4.50 |
| *Staphylococcal enterotoxin C* (m223) | 0 | 0 | 0 | 0.01 | 34.46 |

**Appendix 10. Evaluation of the cross-reactivity of RN-sIgE.**

Considering the high positive rate of RN-sIgE, we must account for potential false positives due to cross-reactivity among different fungal sIgE and SE-sIgE. We first conducted Spearman correlation analyses on 16 fungal sIgE and SE-sIgE pairwise. Results showed that RN-sIgE had weak correlations with other sIgE, with coefficients from 0.10 to 0.29. Semi-quantitative and qualitative analyses showed correlation coefficients between RN-sIgE and other sIgE ranged from 0.06 to 0.24 (semi-quantitative) and from 0.04 to 0.21 (qualitative).


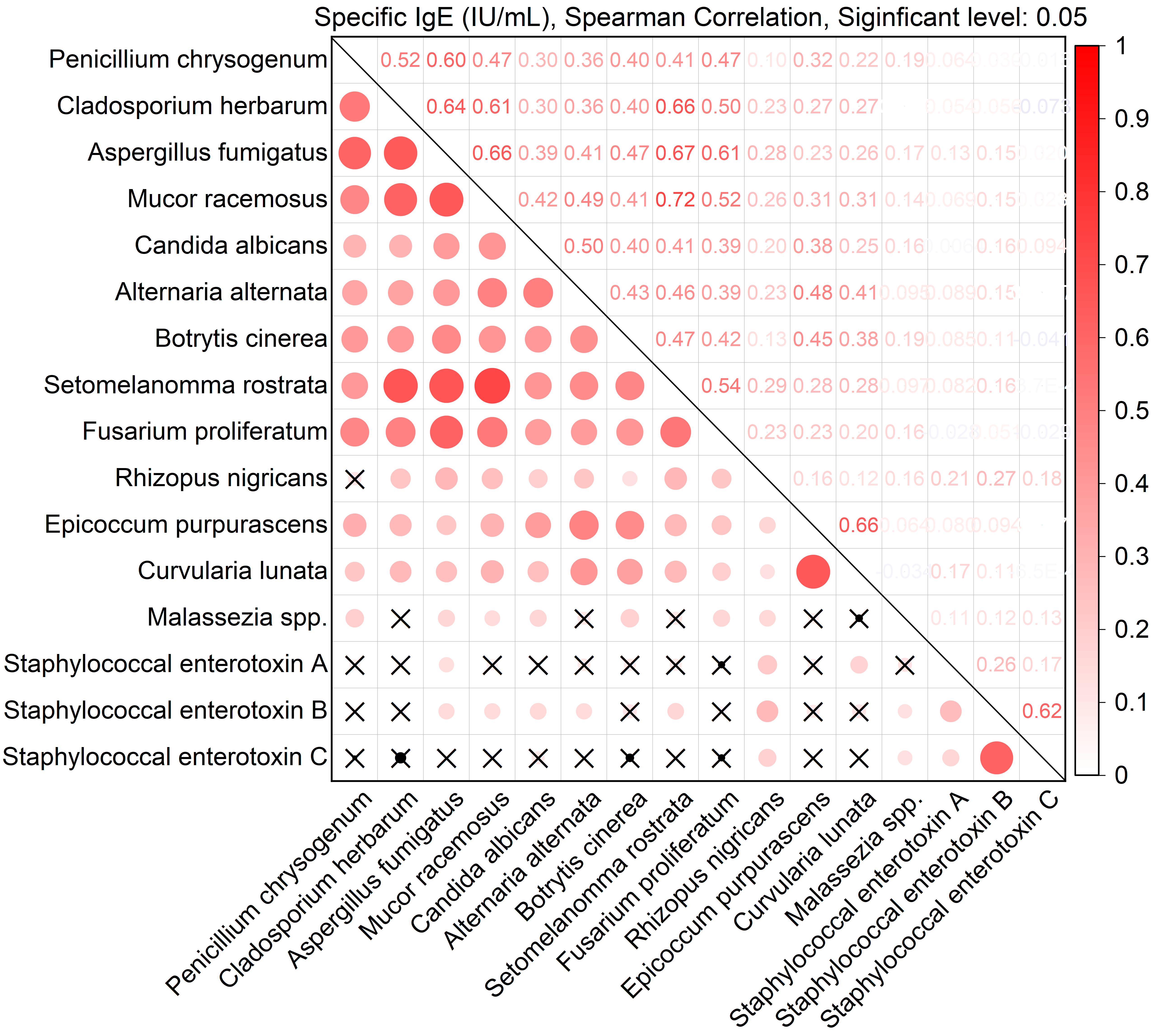


In this study, all patients had allergic asthma. Among them, 24.6% (69/281) tested negative for fungal sIgE and SE-sIgE, and 32.7% (92/281) tested negative for RN-sIgE. Further analysis showed that 39.2% (74/189) of children were sensitized exclusively to RN. Consistency of sIgE results between RN and other fungi and SEs ranged from 33.1% to 52.0%, with a κ coefficient of 0.003 to 0.16, indicating very low consistency. These findings do not support cross-reactivity between RN-sIgE and other fungal sIgE and SE-sIgE.


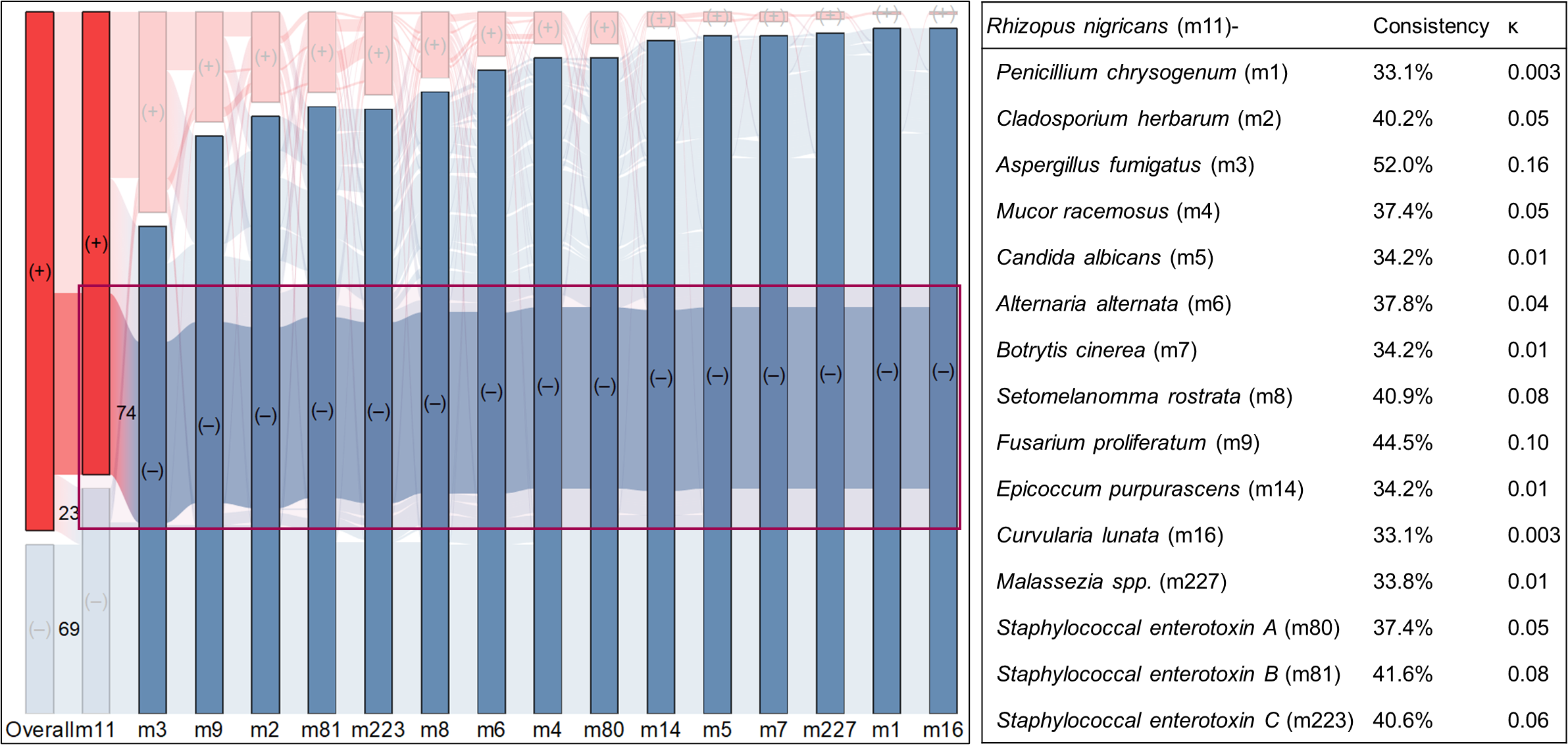


**Appendix 11. Comparison of the RN-sIgE levels between HRTJ and Phadia systems**

A total of 26 children with AA were selected, and serum RN-sIgE levels were re-measured using the Phadia250 system. The results are as follows:

| *Rhizopus nigricans*-sIgE | | |
| --- | --- | --- |
| Patient | HRTJ (IU/mL) | Phadia250 (kU_A_/L) |
| #1 | 4.74 | 0.09 |
| #2 | 2.99 | 0.07 |
| #3 | 2.20 | 0.06 |
| #4 | 1.64 | 0.06 |
| #5 | 2.39 | 0.05 |
| #6 | 2.28 | 0.05 |
| #7 | 1.66 | 0.05 |
| #8 | 0.88 | 0.05 |
| #9 | 2.25 | 0.04 |
| #10 | 1.60 | 0.04 |
| #11 | 0.71 | 0.04 |
| #12 | 0.06 | 0.04 |
| #13 | 0.75 | 0.03 |
| #14 | 0.74 | 0.03 |
| #15 | 0.50 | 0.02 |
| #16 | 0.05 | 0.02 |
| #17 | 0.00 | 0.01 |
| #18 | 0.00 | 0.01 |
| #19 | 0.00 | 0.01 |
| #20 | 12.24 | 1.41 |
| #21 | 10.83 | 0.34 |
| #22 | 9.63 | 0.20 |
| #23 | 8.02 | 0.18 |
| #24 | 6.91 | 0.18 |
| #25 | 5.74 | 0.11 |
| #26 | 5.27 | 0.09 |

The following analysis investigates the discrepancies between the results from the HRTJ and Phadia systems, focusing on four key aspects: instruments, specimens, reading rules, raw materials, and literature sources. The potential causes of the differences are speculated as follows:

**(1) Instrument-related factors**

The Phadia250 system was recalibrated the day before testing and underwent quality control calibration on the day of testing. To further assess the stability of the Phadia250, RN-sIgE levels were retested in four specimens. The results showed 100% qualitative agreement (positive/negative concordance) and a quantitative reproducibility with a correlation coefficient of >0.99, indicating excellent internal stability. Additionally, the Phadia250 successfully performed clinical tests on the same day without issues. For the HRTJ system, each chip underwent internal calibration, as well as negative and positive quality controls, with no problems detected (Appendix 4). These findings indicate that there were no instrument-related issues affecting the results.


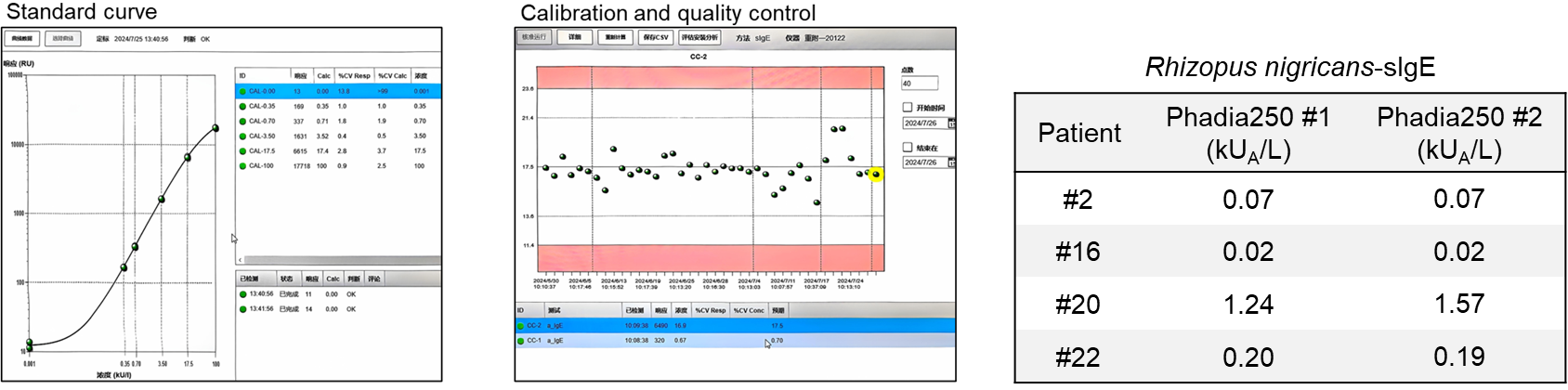


**(2) Specimen-related factors**

The specimens tested using the Phadia250 and HRTJ systems were from the same batch. After aliquoting, they were stored in a biobank without undergoing freeze-thaw cycles. Upon retrieval, they were transported on dry ice, and after thawing overnight at 4°C, they were tested. Existing literature reports that tIgE and sIgE can be preserved for up to two weeks at 4°C and can be stably stored for more than 10 years at -80°C. Additionally, we retested tIgE in two specimens using the Phadia250, and the results were similar to those obtained with the Phadia100 system (which is manually operated; slight discrepancies in results are acceptable). This suggests that there was no degradation of IgE in the specimens.


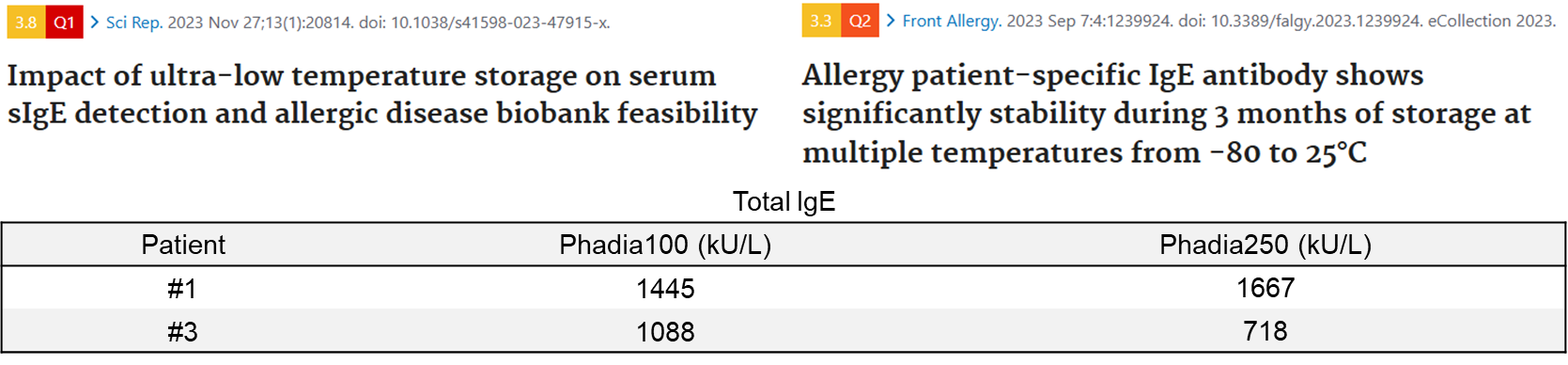


**(3) Reading rules**

The reading rules for the HRTJ and Phadia systems are consistent. During the development phase, results for m1, m2, m3, m5, and m6 were tested in 16 serum specimens using both the HRTJ and Phadia systems, showing an agreement rate of 84.6% and a correlation coefficient of 0.72 (Appendix 3). Furthermore, during the application phase, tIgE levels were tested in 258 serum specimens using both systems, yielding a correlation coefficient of 0.75, which is within the range reported by other studies. These results indicate that there were no issues with inconsistencies in the reading rules between the two systems.

**(4) Raw material-related factors**

The RN used in the HRTJ system was sourced from the internationally renowned allergen raw material supplier, Diagnostische Systeme & Technologien GmbH (DST). DST specializes in allergy and food intolerance diagnostics, producing over 600 high-quality allergens. It holds ISO certification, and its products are primarily sold in Europe and North America, where they are subject to strict market regulations. The RN used in this study came with a quality certificate from DST. In contrast, the raw material for RN in the Phadia system is produced in-house and is not sourced from DST. Moreover, the 2022 EAACI position paper on fungal allergies highlighted the standardization of fungal raw materials as a major technical barrier in fungal allergy research, reflecting the international recognition of this limitation.


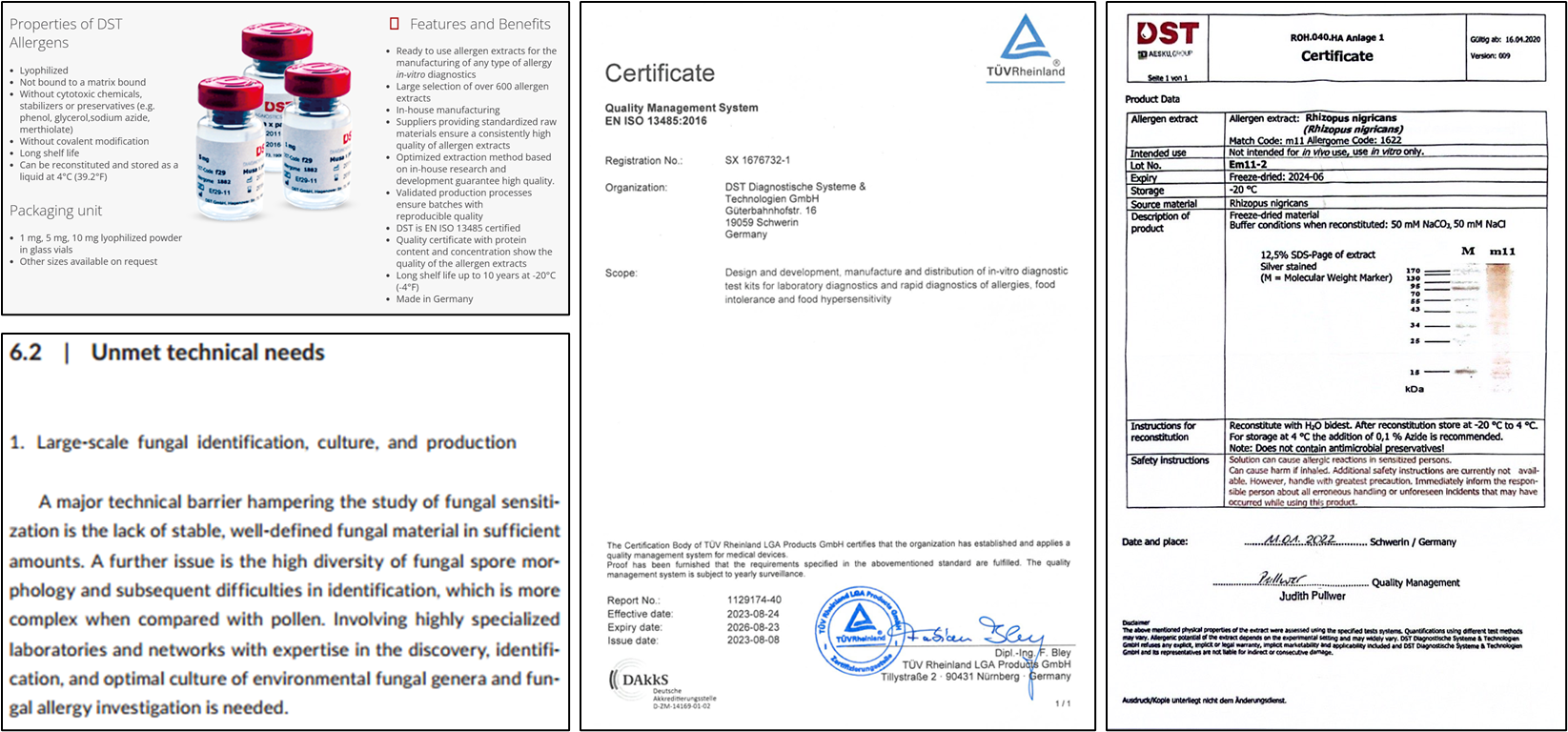


**(5) Literature sources**

Regarding the unexpectedly high sensitization rates to RN in our study, we acknowledge that this finding differs from the majority of existing literature. However, there are studies that report similar observations. For instance, a study conducted in India (Kochar S, Ahlawat M, Dahiya P, Chaudhary D. Assessment of allergenicity to fungal allergens of Rohtak city, Haryana, India. Allergy Rhinol (Providence). 2014;5(2):56-65) found that, although the spore concentration of RN was relatively low, it was still prevalent year-round, indicating that RN is not absent from the air. Furthermore, the study reported a sensitization rate of up to 41.3% in patients with respiratory allergies (see the figure below), which aligns with our results.


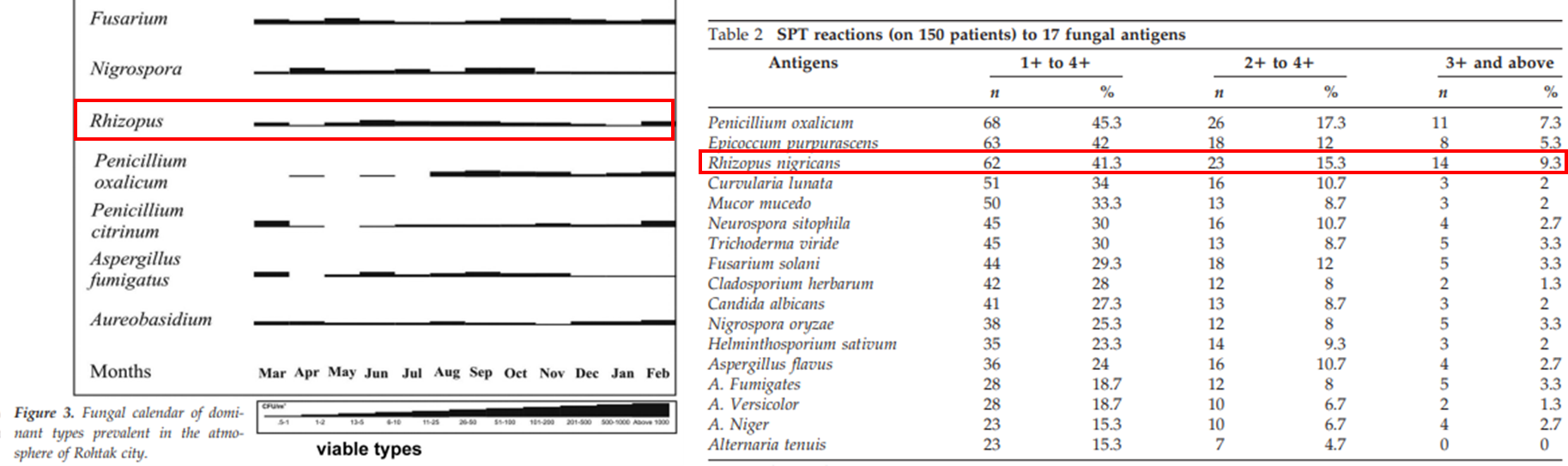


Additionally, a case report presented at the 2016 European Respiratory Society (ERS) Annual Congress (<https://publications.ersnet.org/content/erj/48/suppl60/pa1247>) highlighted a case where RN exposure resulted in significantly elevated total IgE levels, which is consistent with the correlation reported in our study. A study from China (Zhang Y, Sun Y, Liu C, Chen J. Investigation of allergic respiratory system injury in workers exposed to tobacco working environment [in Chinese]. Chinese Journal of Industrial Medicine 2003; 16:9-14) also reported RN-induced lung damage in factory workers, further supporting the relevance of RN in allergic conditions.

**(6) Final analysis and possible explanations**

The analysis of RN-sIgE results from the HRTJ and Phadia250 systems revealed a strong correlation (*Rho* = 0.96, *P* < 0.001). Since fungal extracts typically contain multiple proteins from the species, there may be multiple IgE binding sites. In the absence of clear issues with the instruments, specimens, or reading rules, the observed strong correlation but significant differences in readings could be attributed to differences in IgE binding sites between the two raw materials. Specifically, the DST raw material may contain an important IgE-binding protein, which is present at a much lower concentration in the Phadia raw material. Only this difference could explain the numerical discrepancies observed between the two methods, while still maintaining consistent internal patterns across both systems. Moreover, our bioinformatics analysis revealed differences in the plasma proteome associated with RN sensitization, and these DEPs showed more overlap with mycoses in the UK Biobank (UKB) than with AA.


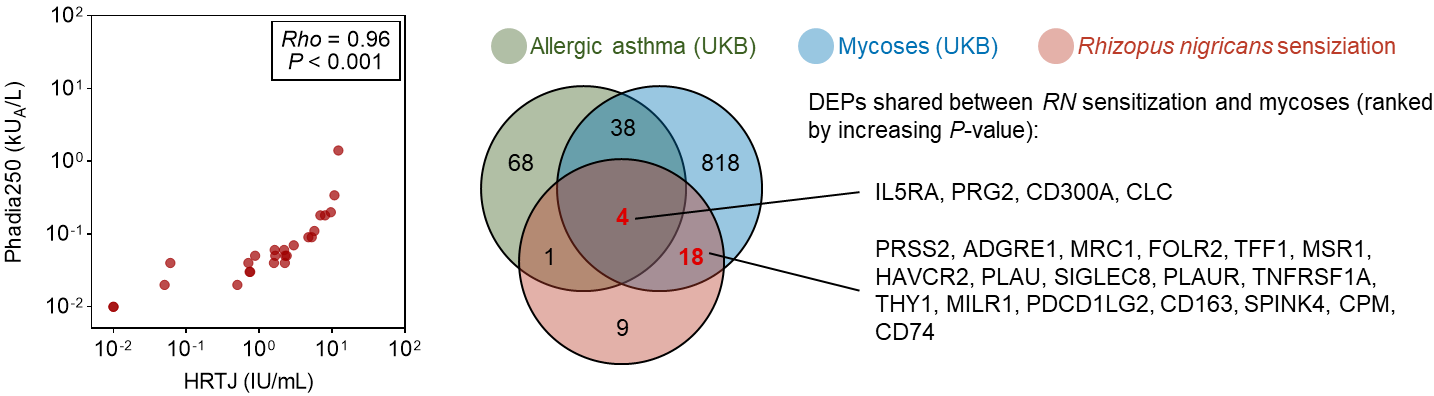


**Appendix 12. Multiple linear regression of sIgE to tIgE.**

| Variable | *β_adj_* (95% CI) | Standardized *β_adj_* | *P* value |
| --- | --- | --- | --- |
| *Aspergillus fumigatus*-sIgE | -267 (-364, -170) | -0.26 | < 0.001 |
| *Rhizopus nigricans*-sIgE | 155 (132, 177) | 0.66 | < 0.001 |
| *Staphylococcal enterotoxin B*-sIgE | 158 (58, 257) | 0.14 | 0.002 |
| The model was adjusted for various characteristics, including age, gender, BMI-for-age Z score, region, family history of allergies, history of allergic rhinitis, tobacco exposure, and asthma medication. The adjusted R² of the model was 0.44 and the Durbin-Watson statistic was 1.78. sIgE: specific IgE; tIgE: total IgE. | | | |

**Appendix 13. Grouping strategy based on RN-sIgE and comparisons of basic characteristics.**


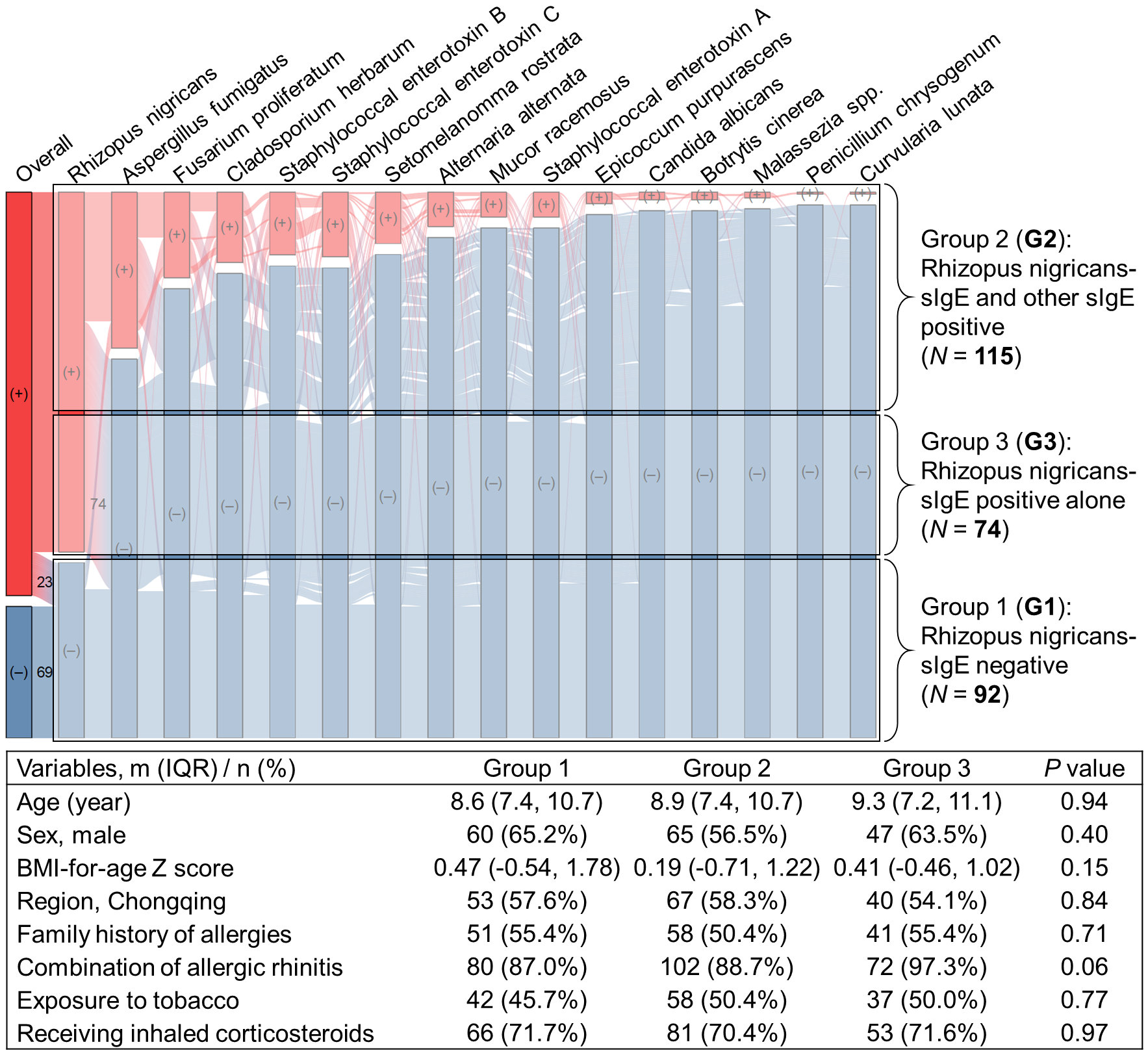


**Appendix 14. The sensitivity analysis for stratified analysis of RN-sIgE.**

C-ACT: Childhood Asthma Control Test; BEC: blood eosinophil count; FeNO: fractional exhaled nitric oxide; sIgE: allergen-specific IgE; RN: *Rhizopus nigricans*. G1: RN-sIgE and other sIgE negative group; G2: RN-sIgE and other sIgE positive group; G3: RN-sIgE positive alone group.


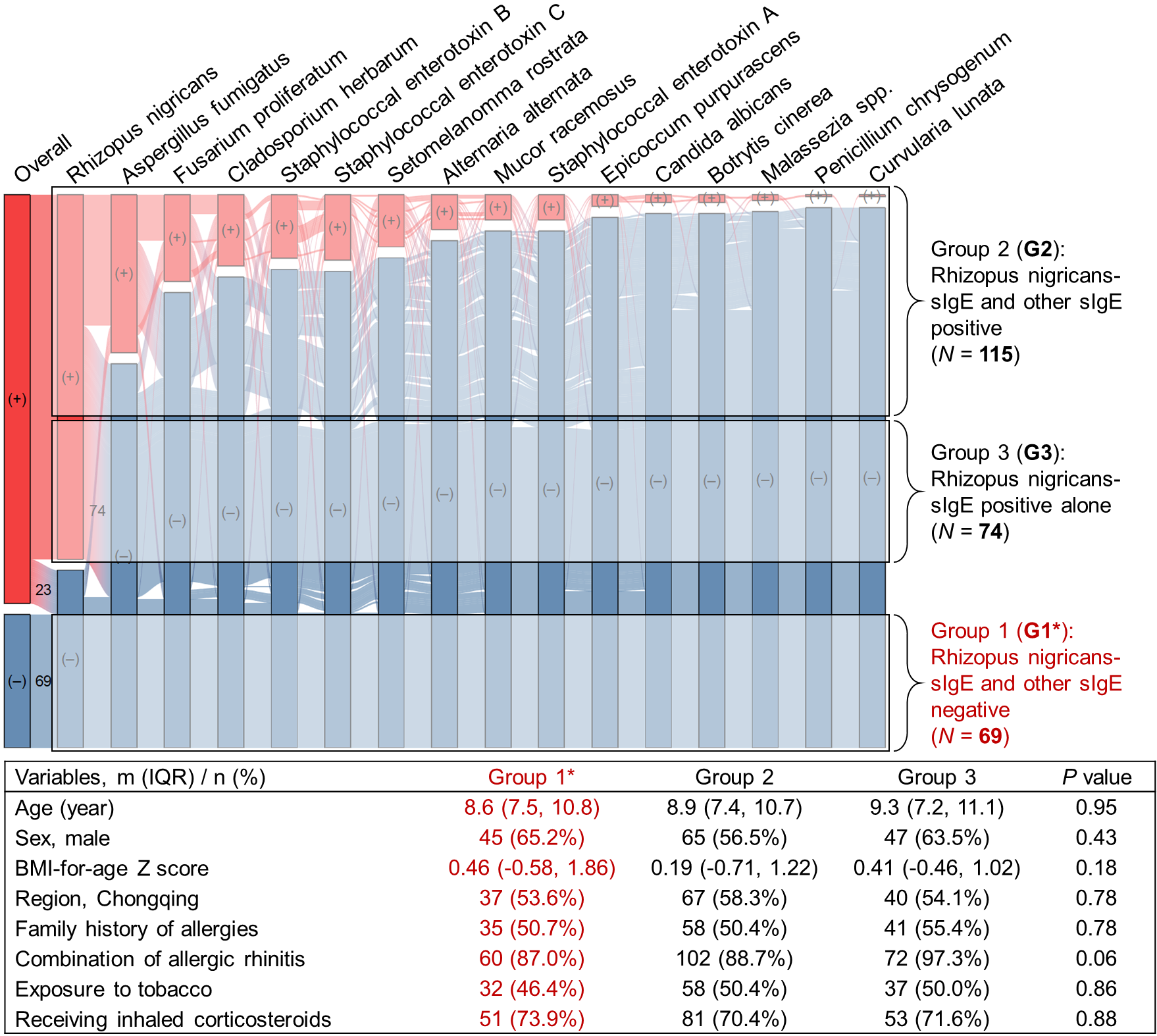


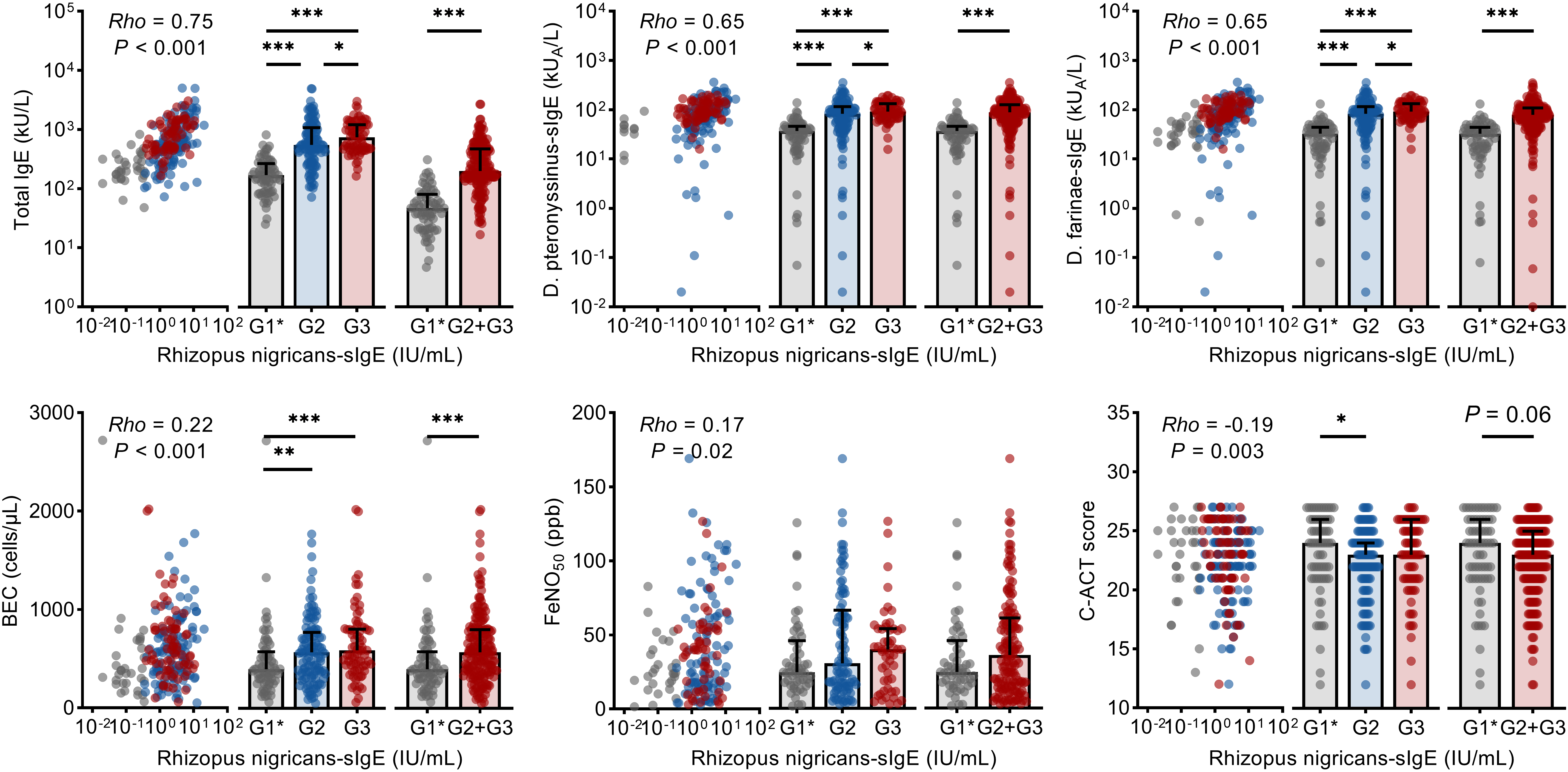


**Appendix 15. The details of the case.**

A 9-year-old boy with acute asthma exacerbation who was diagnosed with asthma 4.4 years ago and used inhaled corticosteroids (ICS) twice daily visited our clinic. In the past month, he experienced daily wheezing and coughing, frequent night awakenings. His C-ACT score was 2 points. He had visited the asthma clinic four times for acute asthma attacks and had a history of AR and severe pneumonia, but no family history of allergies. Physical examination showed stable vital signs, no obesity, and scattered wheezing in both lungs. Due to a recurrent acute asthma attack of unknown cause, the child was admitted for further examination and treatment after outpatient care. Upon admission, tests showed a tIgE level of 461.4 IU/mL (HRTJ system) and an BEC of 1460 cells/μL. sIgE tests for 19 common allergens, including pollen, dust mites, food, and fungi (mx1), were all negative. No significant abnormalities were found in lymphocyte count, other immunoglobulin levels, common respiratory pathogen DNA, or tuberculosis bacillus DNA. A chest CT scan suggested exudative lesions in both lungs. Bronchoscopy revealed chronic suppurative endotracheal and bronchial inflammation, with a significantly increased eosinophil percent (20.0%) in bronchoalveolar lavage fluid.


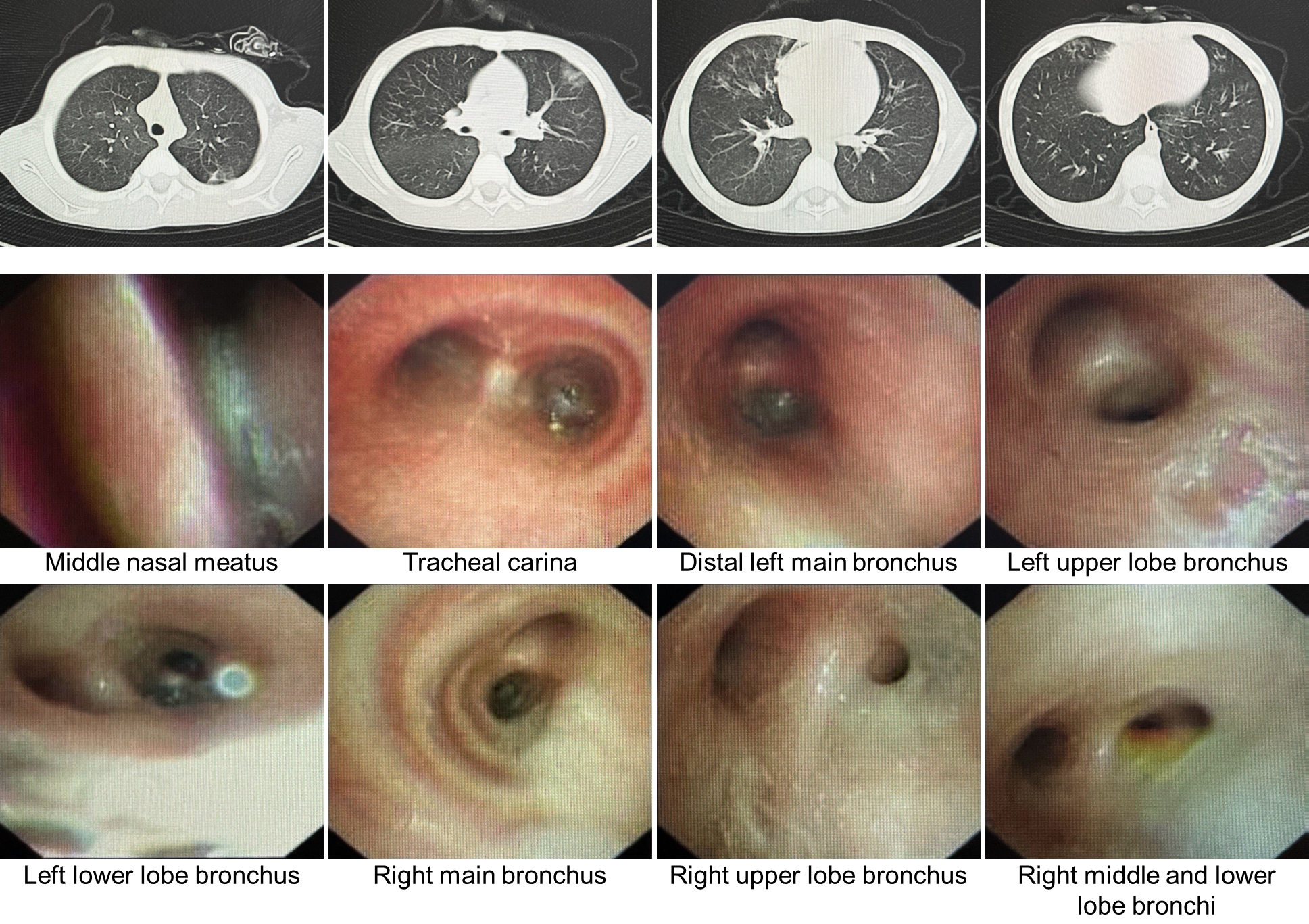


Detection of fungal sIgE and SE-sIgE in the serum samples showed elevated RN-sIgE (0.72 IU/mL) and SEC-sIgE (1.28 IU/mL) levels. A questionnaire about the child’s household environment showed that he lived in a bungalow where coal was used for cooking. The house is damp and prone to mold, with a noticeable mold odor in the kitchen and wardrobe, and frequent sightings of cockroaches and mice. His family rarely cleans curtains, sofas, mattresses, or air conditioning filters, with intervals exceeding six months.
